# Chaihushugan powder regulates the gut microbiota to alleviate mitochondrial oxidative stress in the gastric tissues of rats with functional dyspepsia

**DOI:** 10.3389/fimmu.2025.1549554

**Published:** 2025-02-18

**Authors:** Xuejiao Liu, Keming Yang, Yuebo Jia, Yeliya Yeertai, Chenheng Wu, Xiangxiang Wang, Qingling Jia, Zhijian Gu, Jun Cong, Jianghong Ling

**Affiliations:** ^1^ Department of Gastroenterology, Shuguang Hospital, Shanghai University of Traditional Chinese Medicine, Shanghai, China; ^2^ Department of Gastroenterology, Shenzhen Traditional Chinese Medicine Hospital, Shenzhen, Guangdong, China; ^3^ School of Traditional Chinese Medicine, Shanghai University of Traditional Chinese Medicine, Shanghai, China

**Keywords:** functional dyspepsia, Chinese medicine, gastric dynamics, gut microbiota, fecal microbiota transplantation, oxidative stress

## Abstract

**Introduction:**

Functional dyspepsia (FD) is a prevalent functional gastrointestinal disorder associated with oxidative stress (OS) and dysbiosis. Chaihushugan powder (CHSGP) demonstrates efficacy in treating FD; however, the underlying therapeutic mechanism is not yet elucidated. This study aims to investigate the effects of CHSGP on OS and gut microbiota (GM) in FD rats, with a particular emphasis on the role of GM as a potential target for the antioxidant properties of CHSGP.

**Methods:**

The FD rat model was established with a modified tail-clamp stimulation and the administration of the CHSGP decoction at a dosage of 9.6 g/kg via gavage for a duration of 4 weeks. The GM was depleted by the administration of a cocktail of metronidazole (200 mg/kg), ampicillin (200 mg/kg), neomycin sulfate (200 mg/kg), and vancomycin (100 mg/kg). Fecal microbiota transplantation (FMT) was performed with CHSGP-treated fecal supernatant at a dosage of 10 mL/kg. The gastrointestinal motility was measured using the rates of gastric emptying and small intestine propulsion. Hematoxylin and eosin staining was employed to elucidate the pathological changes, while the transmission electron microscope was used to examine the microstructures of the interstitial cells of Cajal (ICC). Chemiluminescence, colorimetric assay, immunofluorescence co-staining, and western blot assay were employed to identify the OS-related markers (ROS, SOD, NOX4, PRDX1, and TRX2). Sequencing of fecal microbiota was performed utilizing 16S rDNA.

**Results:**

The CHSGP decoction promoted gastrointestinal motility, protected the microstructure of ICC, and reduced OS in FD rats. The GM composition was also regulated by CHSGP. However, these effects disappeared after microbiota depletion. Fortunately, the FMT therapy reinstated them.

**Conclusion:**

Chaihushugan powder decoction might regulate the GM to alleviate mitochondrial OS in the gastric tissues of FD rats.

## 1 Introduction

Functional dyspepsia (FD) is the clinical syndrome characterized by post-meal fullness, premature satiety, epigastric discomfort, or burning. No identifiable structural cause has been found to explain these symptoms (1). According to the Rome criteria, the global prevalence of undiagnosed dyspepsia ranges from 6.9% to 62.8% (2). Patients with FD often suffer from psychological co-morbidity, somatization, low quality of life, and increased financial burdens, alongside an increased burden on social healthcare resources (2). Primary treatments for FD include prokinetic drugs, proton pump inhibitors, *Helicobacter pylori* eradication, neuromodulators, psychotherapy, and probiotics (3). However, treatment outcomes have been unsatisfactory, and patients often experience relapses. Increasing amount of clinical and basic research has shown that traditional Chinese medicine (TCM) is effective for FD (4, 5).

The exact pathogenesis of FD is incompletely understood. Researches have shown that oxidative stress (OS) and gut microbiota (GM) dysbiosis significantly contribute to the pathogenic mechanisms of FD (6, 7). OS refers to the disruption in cellular redox signaling pathways caused by increased concentration of reactive oxygen species (ROS) that exceeds the capability of antioxidant defenses (8). These reactive species cause structural modifications in lipids, proteins, and DNA, potentially resulting in cellular damage (8). The resulting cellular or tissue injury, along with the activation of apoptotic signaling pathways, can subsequently trigger inflammation and contribute to the development of gastrointestinal (GI) mucosal diseases (9). Our previous basic studies confirmed that mitochondrial OS in interstitial cells of Cajal (ICC) caused GI motility disorders in FD (10, 11). On the other hand, gut dysbiosis can lead to impaired duodenal barrier function and low-grade inflammation, which are key mechanisms in the pathogenesis of FD (12). Our prior clinical studies have shown that the diversity and richness of the GM are lower in patients with FD compared to healthy individuals (4). It is worth noting that gut dysbiosis has been recognized as a factor that may affect OS (13). Certain bacteria generate formyl peptides that induce epithelial cell inflammation and elevate intracellular ROS levels (14). Meanwhile, research has demonstrated that probiotics have antioxidative properties and help reduce OS damage (15). Consequently, the pathogenic mechanism of FD may be linked to OS resulting from GM dysregulation. Furthermore, research have demonstrated that drugs exert their effects through GM (16). Numerous studies suggested that the effectiveness of herbal treatments arise from their capacity to influence GM and metabolites (17, 18).

FD is known as “Pi Man” in TCM, with liver qi stagnation identified as the principal reason. TCM theory thinks that Qi is a subtle substance continuously circulating within the human body, playing a vital role in enhancing the functional activities of organs. The function of the liver is to convey and disperse qi and promote its circulation. Consequently, the FD treatment rule is to alleviate liver qi stagnation. Chaihushugan powder (CHSGP) originates from the Ming dynasty, as documented in Jingyue’s Complete Works. It is distinguished remedy that specifically targets liver qi imbalances. This formula has historical significance and has been widely used to manage digestive disorders. It consists of seven traditional plants: *Cyperus rotundus* L. [Cyperaceae], *Conioselinum anthriscoides 'Chuanxiong'* [Apiaceae], *Bupleurum falcatum* L. [Apiaceae], *Paeonia lactiflora Pall.* [Paeoniaceae], *Citrus × aurantium* L. [Rutaceae], *Citrus aurantium f. deliciosa* (Ten.). M. Hiroe [Rutaceae], and *Glycyrrhiza glabra* L. [Fabaceae]. Our preliminary clinical research indicated that CHSGP had considerable effectiveness in the management of FD (4). It effectively improved the dyspepsia symptom scale scores, enhanced the rate of barium strip gastric emptying, and increased the scores of hamilton anxiety scale, hamilton depression scale, and quality of life (4). Additionally, it modulated the composition of GM (4). In fundamental research, CHSGP treatment reduced mitochondrial ROS and serum malondialdehyde (MDA) levels, elevated citrate synthase (CS) and superoxide dismutase (SOD) levels in the gastric tissue, and improved the mitochondrial membrane potential (10, 11). These findings indicated that CHSGP treatment might ameliorate mitochondrial OS and modulate GM in FD rats. However, it remains unclear whether GM is the core target for CHSGP to exert its antioxidant effects. The use of antibiotic cocktails (ABX) significantly reduced the diversity of GM, subsequently impacting cell counts, signaling pathways, and organ functions, with the results resembling those in germ-free animals (19). Consequently, pseudo-sterile animals can be developed utilizing ABX for studies linked to GM depletion (20). Fecal microbiota transplantation (FMT) involves the transfer of healthy microbiota to dysbiotic host to restore beneficial GM and achieve eubiosis (21). FMT can establish the donor microbiota after microbiota depletion using ABX (22). In the majority of TCM research protocols, animals administered herbs or herbal substances for a duration are chosen as FMT donors to explore GM-dependent effects of herbal treatments (18, 23). In this study, we employed ABX and FMT to investigate whether CHSGP treatment could improve mitochondrial OS in the gastric tissues of FD rats by influencing GM.

## 2 Methods and materials

### 2.1 Animals and establishment of the FD rat model

The sample size for the study was established via the KISS approach, as referenced in Festing’s research (24). Power study indicated that a sample size of six rats per group achieved 90% power to identify a 20% change, with a 5% significance level and a two-sided test. Seventy-two male Sprague-Dawley rats, each weighing between 180 and 200 g, were obtained from Shanghai SLAC Animal Biotechnology Co. Ltd. (2017-0005, Shanghai, China). The rats were housed in a specific pathogen-free controlled environment with humidity levels regulated at 55% ± 2%, temperature set at 22°C ± 2°C, and 12-h light/dark cycle. They were offered the unlimited supply of food and water. After a week of adaptation, FD was induced by administering the tail-clamp stimulation for 30 min, twice daily, for 4 weeks (11). Clinical studies found that patients with FD predominantly exhibited the decrease in gastric emptying rate (4) and showed no organic causes upon upper endoscopy (25). Consequently, experimental research utilized the reduction in 24-hour GI transit rate and hematoxylin eosin (HE) staining of gastric tissue, which revealed no pathological changes such as erosion or ulcers, as criteria for FD model establishment (10, 11). The Ethics Committee of the Shanghai University of TCM approved this study on March 17, 2023 (Ethics No. PZSHUTCM2303240005). All animal experimental procedures followed the experimental animal management regulations of the Shanghai University of TCM.

### 2.2 CHSGP preparation and pathway prediction for therapeutic efficacy

CHSGP decoction was composed of the following herbs: 12 g each of *Citrus aurantium f. deliciosa* (Ten.). M. Hiroe [Rutaceae] (20221225, Zhejiang, China) and *Bupleurum falcatum* L. [Apiaceae] (2022121508, Gansu, China); 9 g of *Citrus aurantium* L. [Rutaceae] (20210915, Jiangxi, China), *Ligusticum chuanxiong* [Apiaceae] (2023021907, Sichuan, China), *Paeonia lactiflora Pall*. [Paeoniaceae] (2022121908, Anhui, China), and *Cyperus rotundus* L. [Cyperaceae] (2022102308, Shandong, China); and 3 g of *Glycyrrhiza glabra* L. [Fabaceae] (2023022307, Xinjiang, China) (11). CHSGP decoction was heated in a 37°C water bath and administered to rats via gavage at a dosage of 9.6 g/kg, as previously established (26). We employed ultraperformance liquid chromatography-tandem quadrupole mass spectrometer (UPLC-MS/MS) to analyze and identify CHSGP compounds, subsequently utilizing the CTD and swisstarget databases to determine the action targets of these compounds. Finally, we enriched the cellular components, molecular functions, biological processes, and Kyoto Encyclopedia of Genes and Genomes (KEGG) (KEGG)-related pathways associated with CHSGP using Gene Ontology and the KEGG databases. Relevant results can be found in **Supplementary Tables 1-6**.

### 2.3 Probiotics preparation

The probiotics tablets used were *Bifidobacterium* quadruple viable bacteria tablets (KDJ3YSP, Hangzhou Grand Biologic Pharmaceutical Inc., Hangzhou, China) that were composed of *Bifidobacterium*, *Lactobacillus*, *Enterococcus*, and *Bacillus*. Probiotics were administered at a dosage of 0.945 g/kg following the protocols (27).

### 2.4 Antibiotic cocktail preparation

Consistent with previous studies (28), a combination of four antibiotics, namely metronidazole at 200 mg/kg, ampicillin at 200 mg/kg, vancomycin at 100 mg/kg, and neomycin sulfate at 200 mg/kg, was used to deplete GM. The antibiotics were obtained from Solarbio Science & Technology Co., Ltd. (Beijing, China). The dosage administered was 1 mL per 100 g of body weight.

### 2.5 Fecal microbiota transplantation

We massaged the abdominal region of rats in the FD group treated with CHSGP (FD + CHSGP) to induce defecation and collected the fecal samples using aseptic techniques. Fecal samples were mixed with PBS at a 1:10 ratio, homogenized for 10 min, and centrifuged at 6000 rpm for 15 min. The supernatant was collected and stored at 4°C until transplantation. The administered dosage was 1 ml per 100 g of body mass.

### 2.6 Experimental procedures

We conducted three experiments that used the following protocol:

I. To evaluate the influence of CHSGP decoction on the mitochondrial OS and GM in FD rats, after a week of adaptation, 24 rats were grouped using the randomized block design. The rats were first categorized by body weight, followed by the generation of random numbers through SPSS software to assign them randomly into four groups (n=6): the control group (control), the FD model group (FD), the FD + CHSGP group, and the FD group treated with probiotics (FD + probiotics). FD was induced in all groups except for the control group. FD, FD + CHSGP, and FD + probiotics group rats were administered oral saline, CHSGP decoction, and the probiotics solution, respectively. The dosage was 1.0 mL/100 g given twice daily for 4 weeks. The rats were euthanized under anesthesia on day 29, and samples were collected. The detailed procedure is illustrated in **Supplementary Figure S1A**.

II. To investigate the potential impact of GM on the antioxidant properties of CHSGP, 24 rats were allocated into four groups using the randomized block design (n=6): the control group (control), the group treated with ABX, the group with an ABX-induced FD model [ABX (FD)], and the group with ABX-induced FD treated with CHSGP [ABX (FD + CHSGP)]. After a week of adaptation, the antibiotic cocktail was administered daily via gavage to the ABX, ABX (FD), and ABX (FD + CHSGP) groups for 7 days to induce the pseudo-germ-free state. Subsequently, the ABX (FD) and ABX (FD + CHSGP) groups received saline or the CHSGP decoction via gavage twice daily for 4 weeks concurrent with the tail-clamp simulation. In contrast, we administered saline to the control and ABX groups. On day 36, the rats were euthanized under anesthesia, and the samples were collected. The detailed experimental procedure is illustrated in **Supplementary Figure S1B**.

III. FMT study was conducted to assess the antioxidant properties of CHSGP mediated by GM. Utilizing the randomized block design, 24 rats were divided into four groups (n=6): a control group (control), a group with an FD model (FD), a group with an FD model subjected to ABX-disposed FMT treatment (ABX (FD + FMT)), and a group with an FD model treated with FMT (FD + FMT). From days 1 to 7, the ABX (FD+FMT) group received the ABX intragastrically once a day. From days 8 to 35, the FD and ABX (FD + FMT) groups were administered saline and the CHSGP fecal bacterial solution, respectively, via gavage twice daily, following the 4 weeks of tail-clamp modeling. We administered saline to the control group. The rats were euthanized under anesthesia on day 36, and samples were collected. The detailed procedure is illustrated in **Supplementary Figure S1C**. The timeline for the experimental procedures I-III is illustrated in **Supplementary Figure S2**.

### 2.7 Gastric emptying and intestinal propulsion rate measurements

Semi-solid paste with carbon powder was prepared based on the prior study (10). After the final administration, the rats underwent the fasting duration of 12 h. After 30 min, we anesthetized the rats and opened their abdomens. The stomach and small intestine were excised, and the total weight of the stomach was recorded. The stomach contents were extracted, and the weights of the empty stomachs were recorded. The maximum distance traveled by the black semi-solid paste within the small intestine and the overall length of the small intestine were measured. The gastric emptying rate and the small intestinal propulsion rate were calculated using the following formulas (10): Gastric emptying rate (%) = [1 − (total stomach weight − empty stomach weight)/weight of semi-solid paste] × 100%, and small bowel propulsion rate (%) = furthest distance of carbon powder/total length of small bowel × 100%.

### 2.8 Histopathology

The gastric tissue samples, measuring 3 mm × 3 mm, were preserved in the 4% paraformaldehyde solution. They were dehydrated, coated with paraffin, and sliced into thin sections approximately 4–5 µm thick. After the dewaxing process, the gastric tissue was subjected to hematoxylin and eosin (H&E) staining, dehydrated, penetrated, and encapsulated with neutral resin. The optical microscope (Cx31rtsf, Olympus, China) was employed to examine pathological changes in stomach tissue.

### 2.9 Transmission electron microscopy

The gastric tissues (1 mm^3^) were fixed in the 2.5% glutaraldehyde solution. Ultra-thin sections of 0.5 mm × 0.3 mm were made after rinsing, fixing, rinsing, dehydration, osmosis, and embedding. The morphological features of the ICC were observed after staining using the transmission electron microscope (TEM) (FEI Tecnai G2 Spirit, Czech Republic), emphasizing mitochondrial swelling and vacuoles.

### 2.10 Chemiluminescence

The gastric mitochondria was extracted using the mitochondrial extraction kit (SM0020, Solarbio, China). The mitochondrial supernatant was incubated with a 2’,7’-dichlorodihydrofluorescein diacetate (DCFH-DA) working solution or PBS solution at 37°C for 30 min according to the instructions of the ROS detection kit (E004-1-1, NJJCBIO, China). The wavelength of the enzyme-labeled instrument (SynergyLX, BioTek, Norcross, GA, USA) was set, and the levels of ROS were measured.

### 2.11 Colorimetric assay

The mitochondrial extraction kit (SM0020, Solarbio, China) was utilized to isolate the stomach mitochondria. The mitochondrial supernatant protein was measured using the bicinchoninic acid assay (BCA) kit (P0010S, Beyotime, China). We prepared the substrate application solutions and enzyme working solutions following the instructions provided in the SOD assay kit (A001-3-2, NJJCbio, China). The samples were incubated on plates, and the SOD levels were measured using the enzyme-labeled instrument (SynergyLX, BioTek).

### 2.12 Immunofluorescence co-staining

The histological sections of the gastric tissues were deparaffinized, antigenically repaired using the citrate buffer for 15 min, and blocked with 10% bovine serum albumin (BSA) for 1 h. The sections were incubated overnight at 4°C with primary antibodies NADPH oxidase 4 (NOX4) (1:800, 67681-1-Ig, Proteintech, China), Thioredoxin2 (TRX2) (1:500, 13089-1-AP, Proteintech, China), Peroxiredoxin-1 (PRDX1) (1:800, 66820-1-Ig, Proteintech, China), and Cytochrome oxidase IV (COIV) (1:500, 66110-1-Ig, Proteintech, China). Following the washing steps, the sections were incubated with anti-mouse IgG (A0216, Beyotime, China) and anti-rabbit IgG (A0208, Beyotime, China) at 37°C for 1 h. Following the washing process, the slices were sealed with 4’,6-diamidino-2-phenylindole (DAPI) for 5 min. Finally, the laser confocal microscope (Leica SP8, Germany) was used to examine the samples.

### 2.13 Western blot

Mitochondria from the stomach were obtained using the mitochondrial extraction kit (SM0020, Solarbio, China). The radioimmunoprecipitation assay (RIPA) lysis buffer (P0013E, Beyotime, China) was employed to extract the mitochondrial proteins. After quantification, the proteins were electrophoresed and transferred onto the polyvinylidene fluoride membrane (IPVH00010, Millipore, Ireland). After treatment with 5% skimmed milk, the membranes were co-incubated overnight at 4°C with the following primary antibodies: anti-NOX4 (1:1000, 67681-1-Ig, Proteintech, China), anti-TRX2 (1:500, 13089-1-AP, Proteintech, China), anti-PRDX1 (1:5000, 66820-1-Ig, Proteintech, China), and anti-GAPDH (1:50000, 60004-1-Ig, Proteintech, China). The samples were incubated at room temperature with anti-mouse IgG (1:1000, A0216, Beyotime, China) and anti-rabbit IgG (1:1000, A0208, Beyotime, China) for 1 h. The protein expression was detected using the automated chemiluminescent fluorescence imaging analysis system (5200 Multi, Tanon, China) and quantified with ImageJ software.

### 2.14 GM testing

Rat fecal samples were obtained and stored in liquid nitrogen, held at −80°C until analysis. Genomic DNA from fecal material was extracted from 0.1 g of frozen fecal samples using the E.Z.N.A.^®^ soil DNA Kit (Omega Bio-Tek, Norcross, GA, U.S). The DNA concentration and purity were determined using 1.0% agarose gel electrophoresis and a NanoDrop^®^ ND-2000 spectrophotometer (Thermo Scientific Inc., USA) to ensure that the quality of the samples met the criteria for subsequent analysis. The hypervariable region V3-V4 of the bacterial 16S rRNA gene was amplified with primer pairs 338F (5’-ACTCCTACGGGAGGCAGCAG-3’) and 806R (5’-GGACTACHVGGGTWTCTAAT-3’) (29) using the T100 Thermal Cycler (BIO-RAD, USA). PCR quantification and purification were conducted. Finally, the Illumina NextSeq 2000 PE300 platform (Illumina, San Diego, CA, USA) was selected for high-throughput sequencing and species annotation according to the standard protocols established by Majorbio Bio-Pharm Technology Co. Ltd. (Shanghai, China).

### 2.15 Statistical analysis

Statistical analyses were performed using SPSS version 26.0 and GraphPad Prism version 9.0. The Shapiro-Wilk test employed to assess the normality of the data, and the findings indicated conformity with the normal distribution. The data were presented as means ± standard deviations (x ± SD). Levene’s method was used to test the homogeneity of variances in the data, and the results showed that the variances were homogeneous. Consequently, one-way analysis of variance (ANOVA)was used to compare multiple groups, whereas the Fisher’s least significant difference (LSD) method was used to compare two groups.

The GM structure analysis was based on the α- and β-diversity. The Ace and Chao indices indicated community richness, while the Shannon and Simpson indices assessed community diversity principal coordinate analysis (PCoA), principal component analysis (PCA) and non-metric multidimensional scaling (NMDS) were applicable for comparing species diversity among communities. Additionally, we used the similarity analysis to assess the importance of the detected changes in community structure. The significance level of *p* < 0.05 denoted the significant difference, while a R > 0 indicated that the variation between groups was greater than the variation within groups (30). P values were calculated via one-way ANOVA for multiple group comparisons, and Wilcoxon rank-sum test for two groups comparisons.

## 3 Results

### 3.1 CHSGP promoted gastrointestinal motility in FD rats

**Figure 1:**
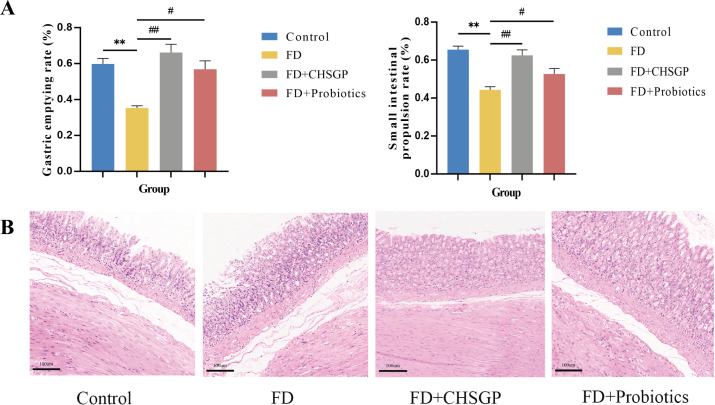
CHSGP promoted the gastrointestinal motility and protected the gastric mucosa in FD rats. **(A)** Test of gastric emptying rate and small intestinal propulsion rate. **(B)** Representative H&E-stained image (200 x magnification). Data were expressed as mean ± standard deviation (x ± SD) (n=6). P values were calculated using One-Way ANOVA followed by Fisher’s LSD test. vs control group, **p < 0.01; vs FD group, #p < 0.05, ##p < 0.01.

FD is characterized by reduced GI motility without any stomach mucosal pathology (11). The results of this study revealed that the FD model, induced by clamp-tail stimulation, exhibited decreased gastric emptying rate (*p* < 0.01) and slower small intestinal movement (*p* < 0.01) compared with the control group, as illustrated in **Figure 1A**. The CHSGP treatment promoted the gastric emptying (*p* < 0.01) and small intestinal propulsion rates (*p* < 0.01). Additionally, H&E staining demonstrated that the gastric tissue glandular structures across all groups were normal and showed no signs of pathological changes such as erosion or ulceration (**Figure 1B**).

### 3.2 CHSGP protected the gastric tissue mitochondria and alleviated OS in FD rats

**Figure 2:**
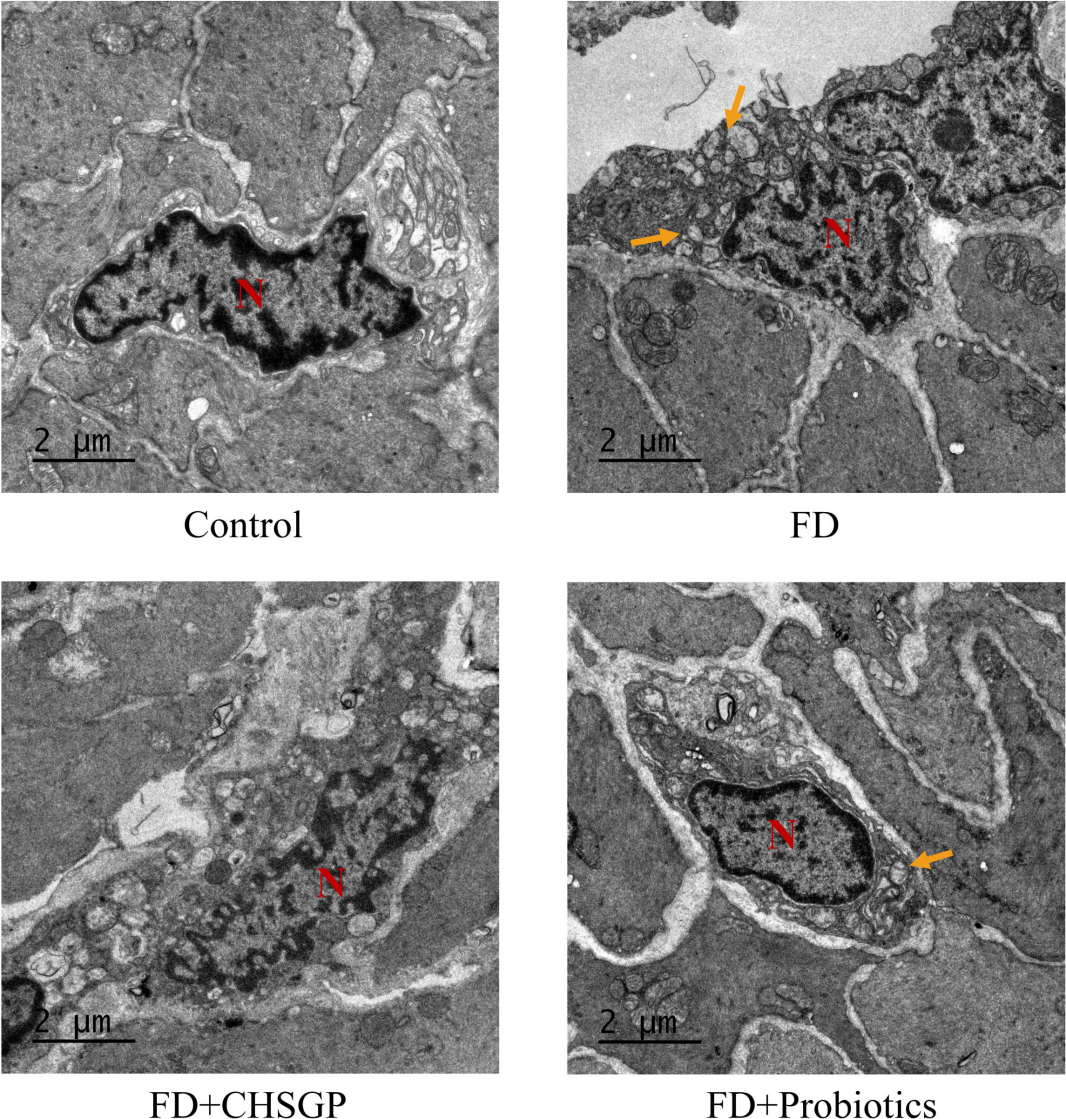
CHSGP protected the gastric tissue ICC mitochondria in FD rats. ICC morphology was observed with a TEM (6000 x magnification). Red N represents the nucleus, and yellow arrows represent the damaged mitochondria.

Wang (11) discovered that the damage of ICC could slow the gastric motility. TEM was used to examine the ultrastructures of the gastric tissue ICC. The results revealed that ICC in the control group had preserved morphologies characterized by elongated or oval shapes, intact nuclear membranes, complete organelles, abundant mitochondria, and clear cytoplasmic structures. Conversely, the FD groups showed noticeable swelling, distortion, and vacuolar changes, accompanied by the absence of mitochondrial cristae. Following the CHSGP treatment, the mitochondria appeared clear and intact with minimal swelling and deformation, as depicted in **Figure 2**.

**Figure 3:**
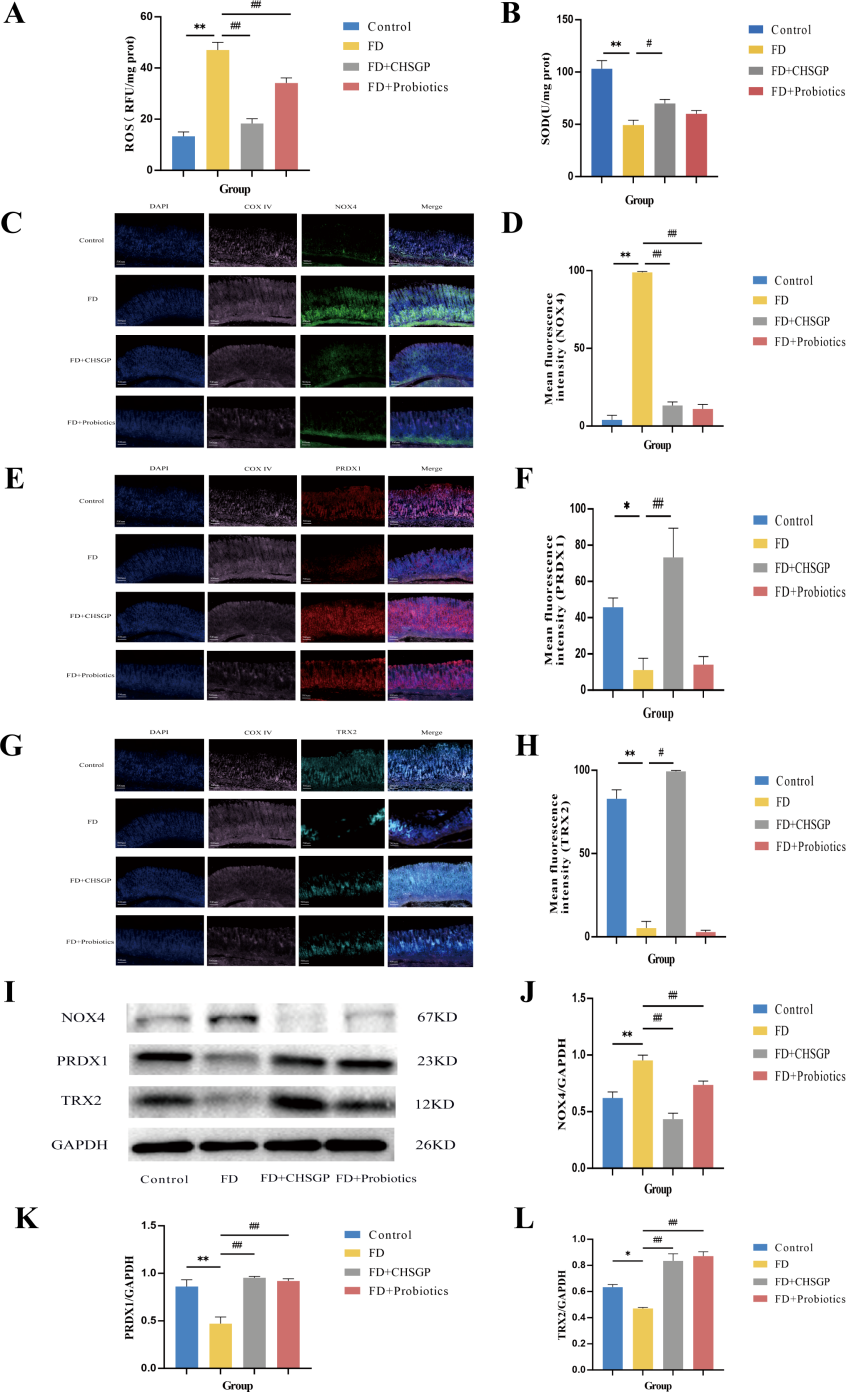
CHSGP alleviated the mitochondrial OS in FD rats. **(A)** ROS content analyzed by chemiluminescence. **(B)** SOD content analyzed by colourimetric assay. **(C)** Staining of gastric tissue with the mitochondrial marker COX IV (pink) and the OS marker NOX4 (green). **(D)** Mean fluorescence intensity of NOX4. **(E)** Staining of gastric tissue with the mitochondrial marker COX IV (pink) and the OS marker PRDX1 (red). **(F)** Mean fluorescence intensity of PRDX1. **(G)** Staining of gastric tissue with the mitochondrial marker COX IV (pink) and the OS marker TRX2 (turquoise). **(H)** Mean fluorescence intensity of TRX2. **(I)** The protein expression levels of NOX4, PRDX1 and TRX2 were analyzed by WB. **(J-L)** Density calculations for NOX4, PRDX1 and TRX2. Data were expressed as mean ± standard deviation (x ± SD) (n=6). P values were calculated using One-Way ANOVA followed by Fisher’s LSD test. vs control group, *p < 0.05, **p < 0.01; vs FD group, ^#^ *p* < 0.05, ^##^ *p* < 0.01.

The ultrastructure of mitochondria can be compromised by OS (31). The gastric mitochondria were analyzed for levels of ROS, SOD, NOX4, PRDX1, and TRX2 related to OS. The findings indicated that the FD group had increased mitochondrial ROS (*p* < 0.01) and reduced SOD (*p* < 0.01) compared with the control group (**Figures 3A, B**). After CHSGP treatment, the levels of mitochondrial ROS were decreased (*p* < 0.01), whereas SOD levels were increased (*p* < 0.05) (**Figures 3A, B**). The levels of mitochondrial OS were assessed by the immunofluorescence co-localization method for relative quantification. The mitochondrial marker CoxIV (pink) was co-localized with the OS markers NOX4 (green), PRDX1 (red), and TRX2 (turquoise). The co-localized expression of NOX4 and COXIV was significantly elevated in the FD group compared with the control group (*p* < 0.01), as shown in **Figures 3C, D**. Conversely, the co-localized expression of PRDX1 (*p* < 0.05) or TRX2 (*p* < 0.01) with COXIV exhibited a significant decrease, as shown in **Figures 3E-H**. CHSGP intervention reversed the above results. Notably, the co-localized expression of NOX4 and COXIV was markedly reduced (*p* < 0.01), while the expression levels of PRDX1 (*p* < 0.01) or TRX2 with COXIV had risen (*p* < 0.05) (**Figures 3C-H**). Additionally, the western blot (WB) analysis revealed that the FD group had elevated levels of the NOX4 protein (*p* < 0.01) and decreased levels of the PRDX1 (*p* < 0.01) and TRX2 proteins (*p* < 0.05) compared with the control group, as illustrated in **Figures 3I-L**. Following the administration of CHSGP decoction, NOX4 protein levels decreased (*p* < 0.01), while PRDX1 (*p* < 0.01) and TRX2 (*p* < 0.01) protein levels increased (**Figures 3I-L**). These findings indicated that CHSGP treatment ameliorated mitochondrial OS damage.

### 3.3 CHSGP regulated the diversity and composition of GM in FD rats

**Figure 4:**
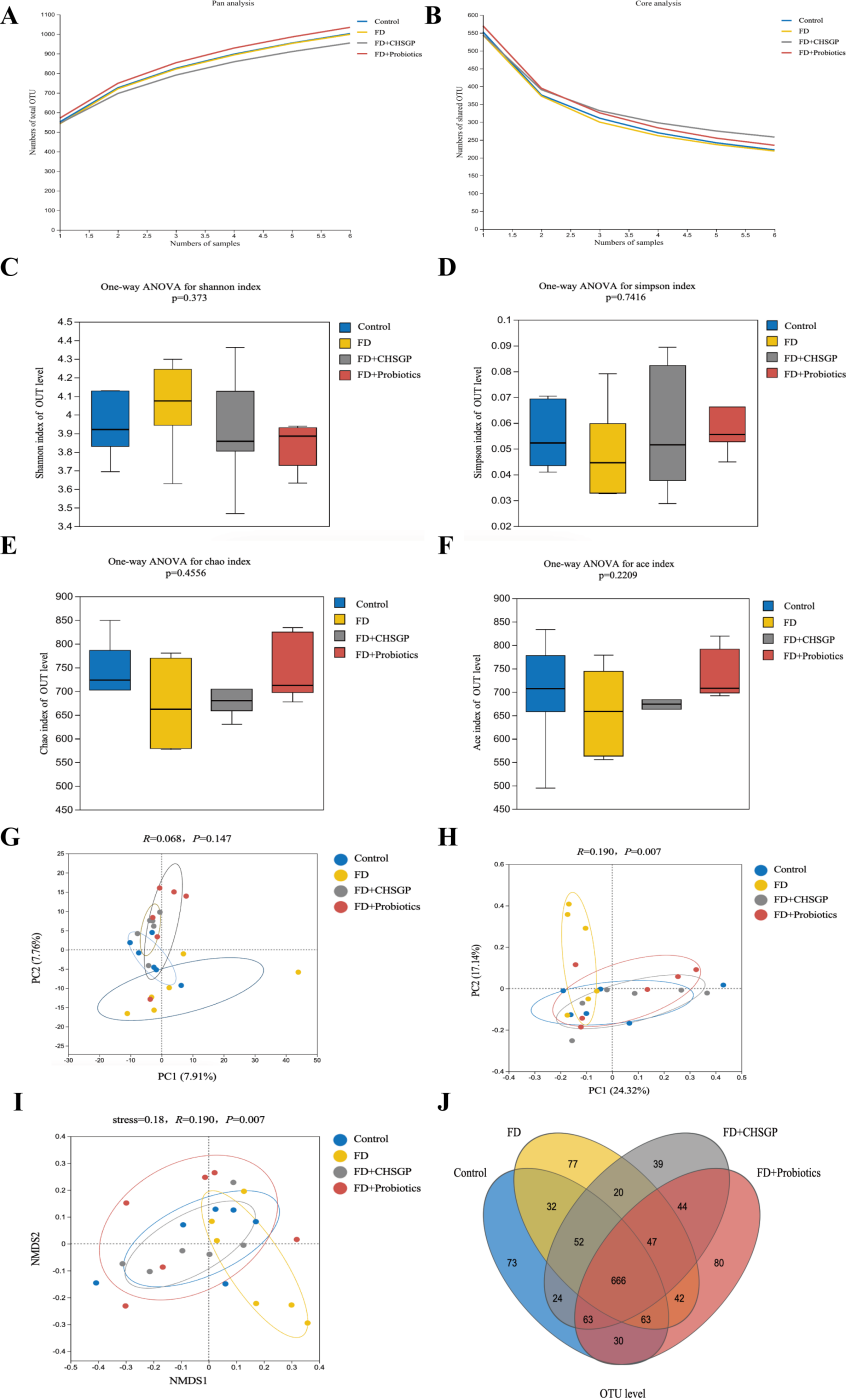
CHSGP regulated the diversity of the GM in FD rats. **(A, B)** Pan/Core curve. **(C-F)** Alpha diversity analysis. Data were expressed as mean ± standard deviation (x ± SD) (n = 6). Differences between samples were compared using the ANOSIM test. **(C)** Shannon index analysis. **(D)** Simpson index analysis. **(E)** Chao index analysis. **(F)** Ace index analysis. **(G-I)** Beta diversity analysis. Data were expressed as mean ± standard deviation (x ± SD) (n = 6). Differences between samples were compared using the ANOSIM test. **(G)** PCA analysis. **(H)** PCOA analysis. **(I)** NMDS analysis. **(J)** OTU analysis: Venn diagrams.

High-throughput gene sequencing analysis of 16S rDNA extracted from the rat fecal microbiota DNA was performed to evaluate the impact of the CHSGP therapy on the microbial community. The pan/core curve illustrated the common or core species found within the sample. The sequencing curve reached a plateau, suggesting that it was satisfactory for capturing the complete spectrum of species in the analyzed samples (**Figures 4A, B**). Subsequent analysis that used the existing data was considered appropriate. α- and β-diversity analyses were performed to assess the variability within GM. Several operational taxonomic unit (OTU)-based indices, including the Simpson, Shannon, Chao, and Ace indices, showed that no notable differences among the four groups (*p* > 0.05). This indicated similar microbial abundance and homogeneity in the α-diversity analysis (**Figures 4C-F**). The β-diversity of the microbiota was illustrated by the PCoA, PCA, and NMDS analyses. The PCA dissimilarities across the groups were assessed using the analysis of similarities, and PCoA was performed using the Bray-Curtis distance metric. The PCA showed no statistical difference among the four groups of GM (*p* > 0.05) (**Figure 4G**); however, the PCoA indicated statistical significance among the groups (*p* < 0.01) (**Figure 4H**). Therefore, the FD group exhibited the distinct clustering pattern in comparison to the control group, indicating that the structural composition of the sample was dissimilar (**Figure 4H**). Furthermore, the bacterial composition of the CHSGP group exhibited greater similarity to that of the control group (**Figure 4H**). Researchers have used NMDS analyses to transform entities from the multidimensional space into the lower-dimensional space. This contributes to their positioning, analysis, and classification. It also preserves the original relationships between the objects. We tested the NMDS analysis by measuring the stress value. The results showed that GM of the FD group significantly differed from those of the control group (*p* < 0.01) (**Figure 4I**). Furthermore, the GM composition of the CHSGP group was more similar to that of the control group. Next, we used Venn diagrams to analyze the differences in the number of OUTs for the four sample communities. A total of 578 OTUs were identified as common across all groups. Specifically, the control group exhibited 772 OTUs, while the FD group contained 737 OTUs. The FD + CHSGP and FD + probiotics groups each included 740 OTUs (**Figure 4J**).

**Figure 5:**
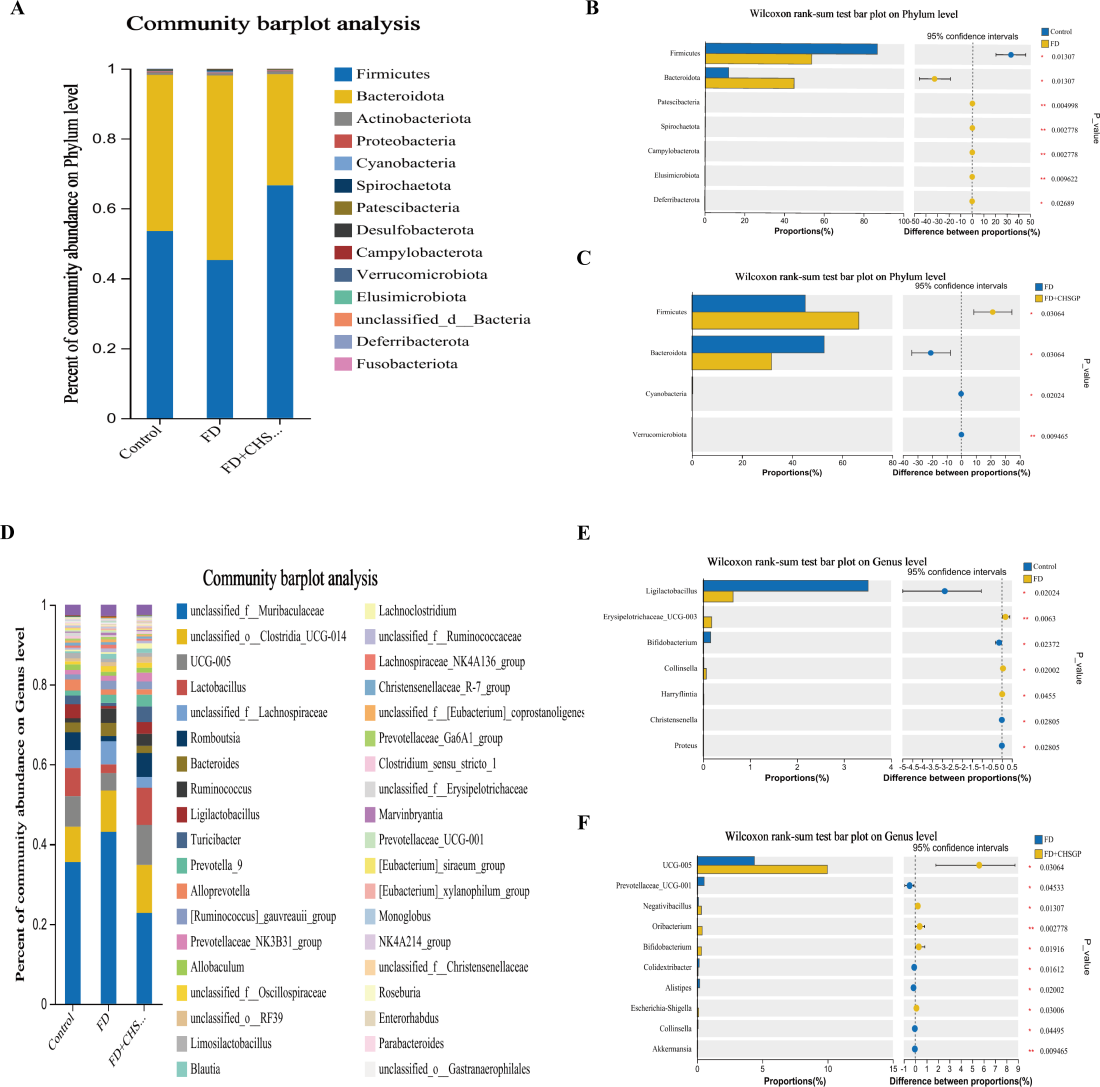
CHSGP regulated the species composition of the GM in FD rats. **(A)** Columnar stack diagram of species composition at the phylum level between the control, FD, and FD + CHSGP groups. **(B)** Differential strains at the phylum level between control and FD groups. **(C)** Differential strains at the phylum level between FD and FD + CHSGP groups. **(D)** Columnar stack diagram of species composition at the genus level between the control, FD, and FD + CHSGP groups. **(E)** Differential strains at the genus level between control and FD groups. **(F)** Differential strains at the genus level between FD and FD + CHSGP groups. Data were expressed as mean ± standard deviation (x ± SD) (n=6). P values were calculated using Wilcoxon rank-sum test. *p < 0.05, **p < 0.01.

The species compositions of the control, FD, and FD + CHSGP groups were analyzed at the phylum and genus levels. Fourteen bacterial categories were identified at the phylum level, with Firmicutes and Bacteroidetes being the most common, constituting roughly 90% of the total bacterial population (**Figure 5A**). Compared with the control group, the FD group had a rise in Bacteroidetes (*p* < 0.05) and a decline in Firmicutes (*p* < 0.05). This result indicated the disrupted microbiota (**Figure 5B**). CHSGP treatment restored the microbial composition of the FD. Compared with the FD group, the CHSGP group had reduced Bacteroidetes (*p* < 0.05) and increased Firmicutes (*p* < 0.05) (**Figure 5C**).

At the genus level, 38 bacteria were identified. They primarily included *norank_f_Muribaculaceae*, *unclassified_o:Clostridia_UCG-014*, *UCG-005*, *Lactobacillus*, *unclassified_f:Lachnospiraceae*, *Romboutsia*, and *Bacteroides* (**Figure 5D**). The FD group had reduced levels of *Ligilactobacillus* (*p* < 0.05) and *Bifidobacterium* (*p* < 0.05) while higher levels of *Erysipelotrichaceae_UCG-003* (*p* < 0.01) and *Collinsella* (*p* < 0.05) compared to the control group (**Figure 5E**). After the CHSGP treatment, there was a decrease in the proportions of *Prevotellaceae_UCG-001* (*p* < 0.05), *Colidextribacter* (*p* < 0.05), *Alistipes* (*p* < 0.05), and *Akkermansia* (*p* < 0.01), while the proportions of *UCG-005* (*p* < 0.05), *Oribacterium* (*p* < 0.01), *Negativibacillus* (*p* < 0.05), and *Bifidobacterium* (*p* < 0.05) were raised compared with the FD group (**Figure 5F**).

### 3.4 CHSGP regulated the GM to alleviate OS

**Figure 6:**
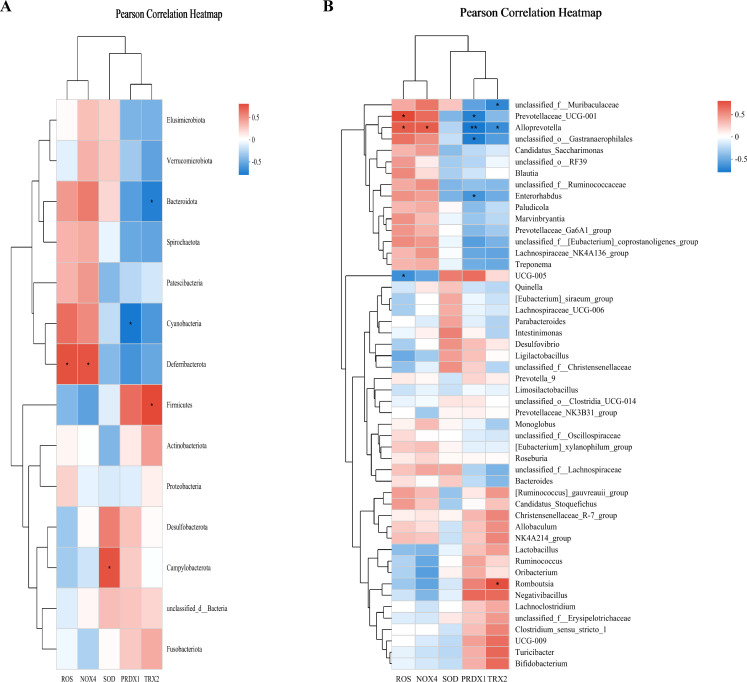
CHSGP regulated the GM to alleviate OS. **(A)** Heatmap of the correlation between the differential microbiota at the phylum level among the control group, FD group, and FD + CHSGP group and the indicators of OS. **(B)** Heatmap of the correlation between the differential microbiota at the genus level among the control group, FD group, and FD + CHSGP group and the indicators of OS. We calculated the correlation between variables using the pearson correlation coefficient. *p < 0.05, **p < 0.01.

Pearson’s correlation analysis was employed to evaluate the relationship between GM and the indicators of OS (ROS, SOD, NOX4, TRX2, and PRDX1) across the control, FD, and FD + CHSGP groups. At the phylum level, Bacteroidota exhibited negative correlation with TRX2 (*p* < 0.05), while Cyanobacteria had negative correlation with PRDX1 (*p* < 0.05). Conversely, Deferribacterota exhibited positive correlation with ROS (*p* < 0.05) and NOX4 (*p* < 0.05), Firmicutes showed positive correlation with TRX2 (*p* < 0.05), and Campylobacterota shown positive correlation with SOD (*p* < 0.05) (**Figure 6A**). At the genus level, *Prevotellaceae-UCG-001* exhibited positive correlation with ROS (*p* < 0.05), while negative correlation with PRDX1 (*p* < 0.05). *Alloprevotella* showed positive correlations with ROS (*p* < 0.05) and NOX4 (*p* < 0.05), but it had negative correlations with PRDX1 (*p* < 0.01) and TRX2 (*p* < 0.05). *UCG-005* correlated negatively with ROS (*p* < 0.05). *Unclassified-o-Gastranaerophilales* and *Enterohabdus* exhibited negative correlation with PRDX1 (both *p* < 0.05). *Romboutsia* had positive correlation with TRX2 (*p* < 0.05), while *unclassified-f-Muribaculaceae* demonstrated negative correlation with TRX2 (*p* < 0.05) (**Figure 6B**).

### 3.5 CHSGP relied on the GM to promote gastrointestinal motility in FD rats

#### 3.5.1 Gastrointestinal motility changes in FD rats after ABX treatment

**Figure 7:**
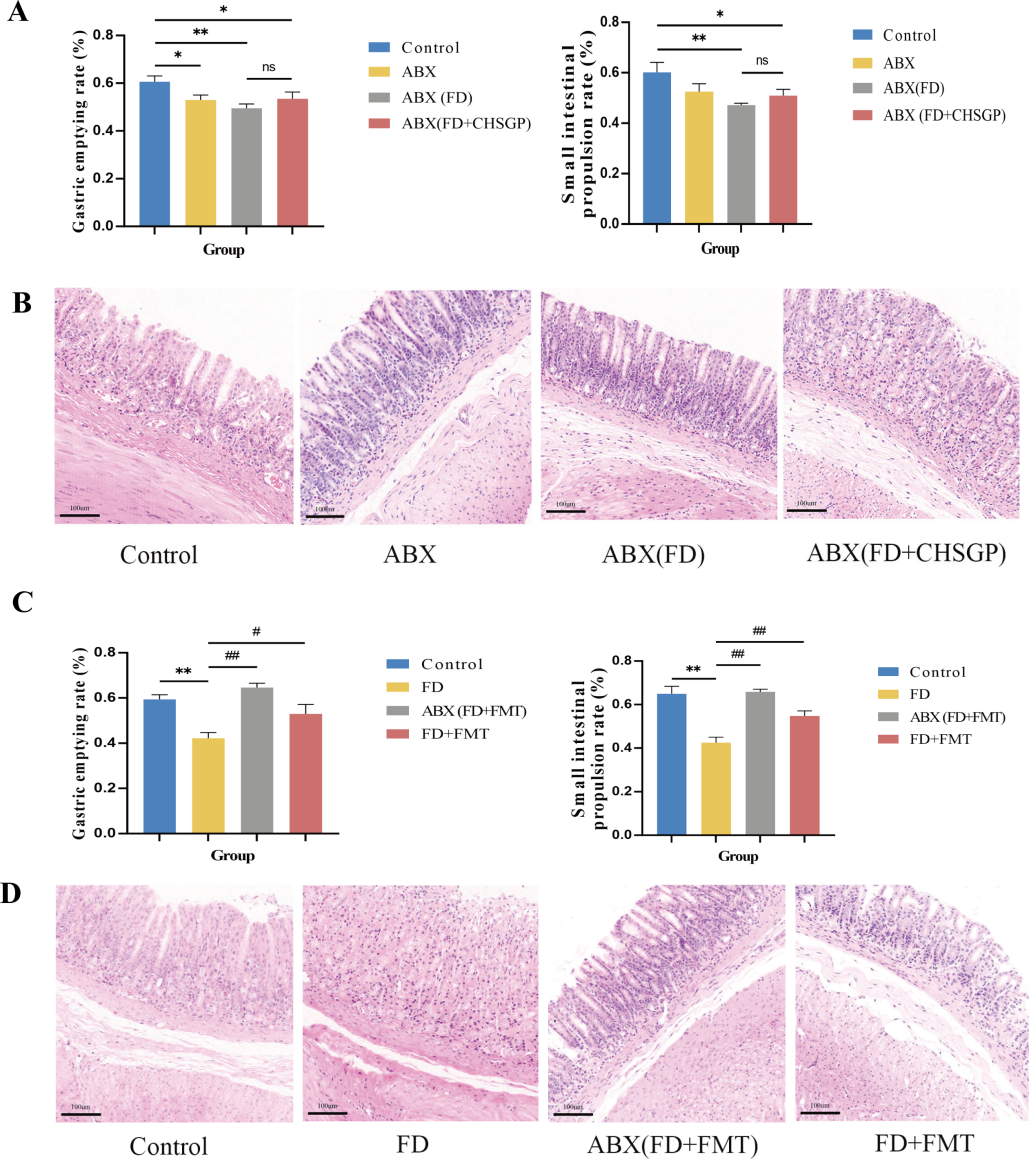
CHSGP relied on the GM to promote gastrointestinal motility in FD rats. **(A-B)** Changes of the gastrointestinal motility and gastric mucosa in FD after ABX: **(A)** Test of the gastric emptying rate and small intestinal propulsion rate. **(B)** Representative H&E-stained image (200 × magnification). **(C, D)** Changes of the gastrointestinal motility and gastric mucosa in FD after FMT: **(C)**Test of the gastric emptying rate and small intestinal propulsion rate. **(D)** Representative H&E-stained image (200 x magnification). Data were expressed as mean ± standard deviation (x ± SD) (n=6). P values were calculated using One-Way ANOVA followed by Fisher’s LSD test. vs control group, *p < 0.05, **p < 0.01; ABX (FD) vs ABX (FD + CHSGP) group, ns*p* > 0.05; vs FD group, ^#^ *p* < 0.05, ^##^ *p* < 0.01.

The rats were administered ABX to deplete their GM seven days prior to experiment II. The ABX group exhibited a notably slower gastric emptying rate than the control group (*p* < 0.05). The ABX (FD) and ABX (FD + CHSGP) groups had lower gastric emptying rates (*p* < 0.01 and *p* < 0.05, respectively) and slower small intestinal propulsion rates (*p* < 0.01 and *p* < 0.05, respectively) than the control group (**Figure 7A**). However, no statistically significant differences were seen between the ABX (FD) and ABX (FD + CHSGP) groups (both *p* > 0.05) (**Figure 7A**). The H&E staining showed structurally intact gastric tissues in the ABX, ABX (FD), and ABX (FD + CHSGP) groups, without any signs of organic damage such as erosion or ulcers (**Figure 7B**). However, a slight neutrophil infiltration was observed in the mucosal layer. The aforementioned findings suggested that GM depletion led to the disappearance of GI propulsion mediated by CHSGP.

#### 3.5.2 Gastrointestinal motility changes in FD rats after FMT treatment

The rat fecal samples treated with FD + CHSGP were collected early and stored at −80°C. The fecal bacterial solution was transferred to rats with or without depleted GM. After the FMT, the results showed that the ABX (FD + FMT) and FD + FMT groups exhibited increased gastric emptying rates (*p* < 0.01 and *p* < 0.05, respectively) and small intestinal propulsion rates (both *p* < 0.01) compared with the FD group (**Figure 7C**). Additionally, the HE sections showed no structural damage or organic lesions in any group (**Figure 7D**). Therefore, CHSGP relied on the GM to enhance GI motility in FD.

### 3.6 CHSGP relied on GM to protect ICC’s mitochondria in FD rats

#### 3.6.1 Mitochondrial ultrastructure changes in FD rats after ABX treatment

**Figure 8:**
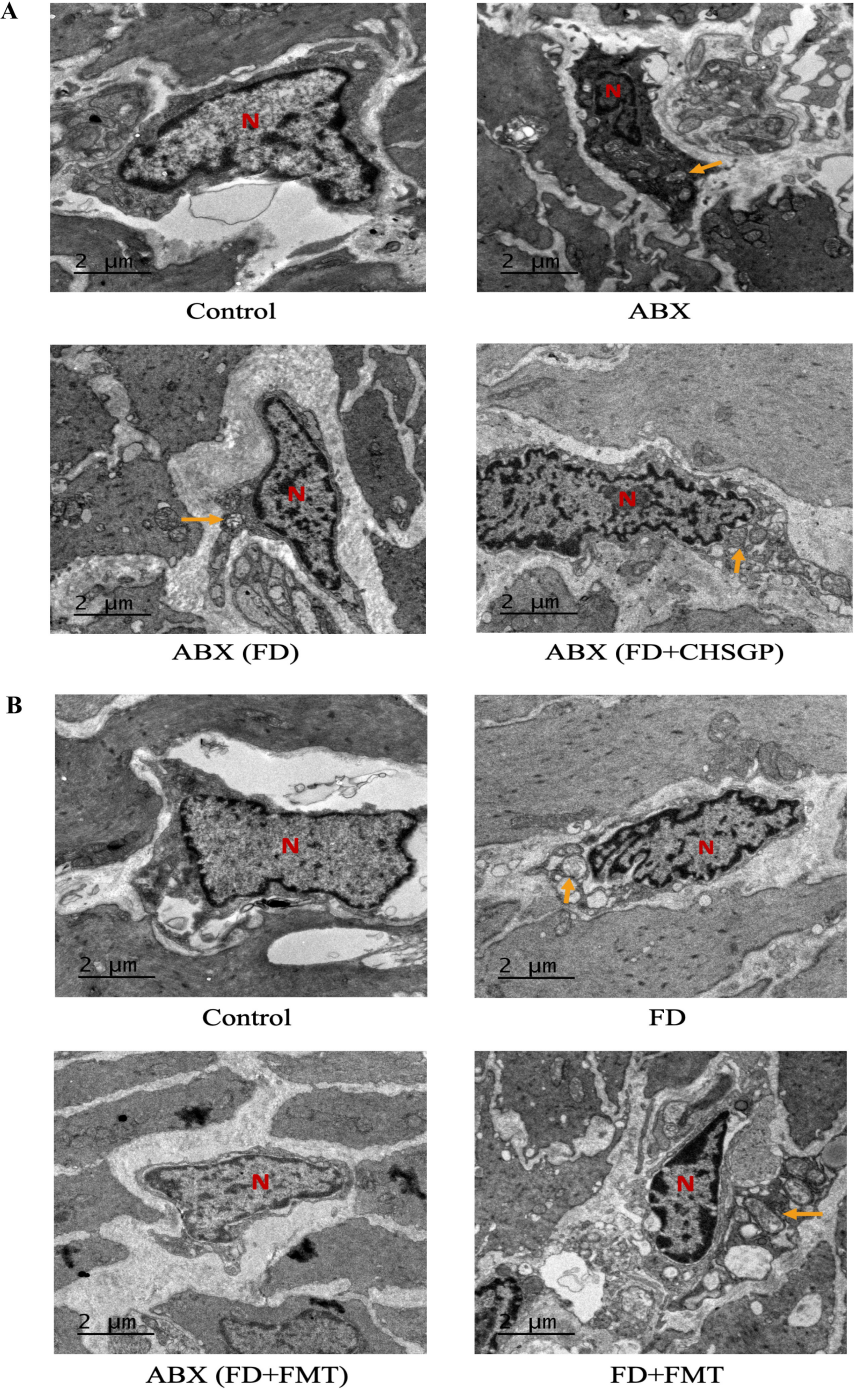
CHSGP relied on the GM to protect ICC mitochondria in FD rats. **(A)** ICC morphology was observed with a TEM (6000 x magnification) after ABX. **(B)** ICC morphology was observed with a TEM (6000 x magnification) after FMT. Red N represents the nucleus, and yellow arrows represent the damaged mitochondria.

The TEM images revealed significant swelling, distortion, and vacuolization in the mitochondria of ICC following the antibiotic treatment. This was accompanied by the disappearance of cristae in the groups treated with ABX, ABX (FD), and ABX (FD + CHSGP) (**Figure 8A**). This indicated that the protective role of CHSGP in mitochondria was lost after GM depletion. As a result, GM contributed to the protective effect of CHSGP on the mitochondria.

#### 3.6.2 Mitochondrial ultrastructure changes in FD rats after FMT treatment

Mitochondria of the ICC showed swelling and deformities in the FD + FMT group after the FMT treatment, while they remained clear and intact in the ABX (FD + FMT) group (**Figure 8B**). These results indicated that the CHSGP fecal microbiota offered specific protection against the mitochondrial damage resulting from dysbiosis of microbiota in FD.

### 3.7 CHSGP relied on GM to alleviate mitochondrial OS in FD rats

#### 3.7.1 Mitochondrial OS changes in FD rats after ABX treatment

**Figure 9:**
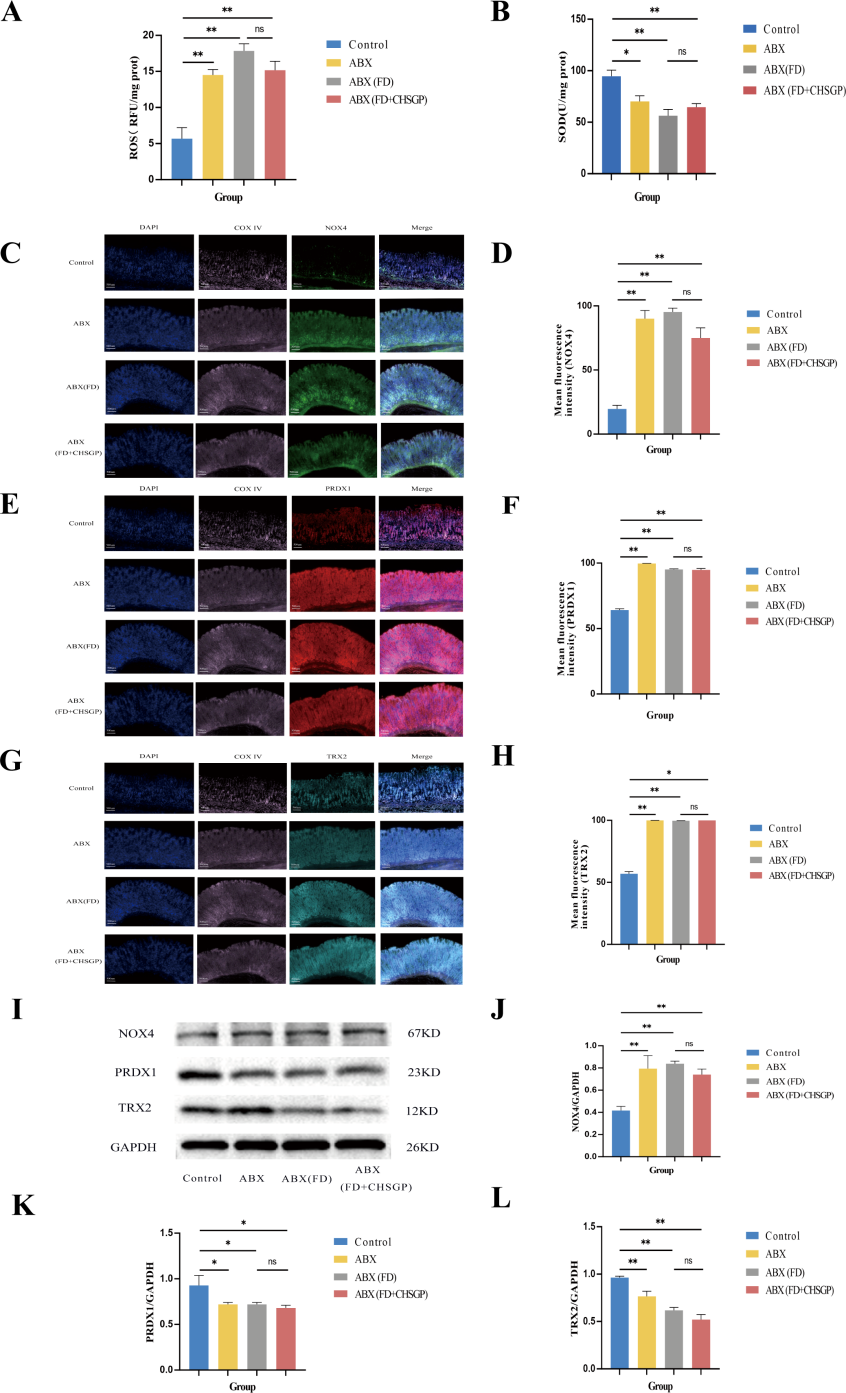
Changes of the mitochondrial OS in FD rats after ABX. **(A)** ROS content analyzed by chemiluminescence. **(B)** SOD content analyzed by colourimetric assay. **(C)** Staining of gastric tissue with the mitochondrial marker COX IV (pink) and the OS marker NOX4 (green). **(D)** Mean fluorescence intensity of NOX4. **(E)** Staining of gastric tissue with the mitochondrial marker COX IV (pink) and the OS marker PRDX1 (red). **(F)** Mean fluorescence intensity of PRDX1. **(G)** Staining of gastric tissue with the mitochondrial marker COX IV (pink) and the OS marker TRX2 (turquoise). **(H)** Mean fluorescence intensity of TRX2. **(I)** The protein expression levels of NOX4, PRDX1 and TRX2 were analyzed by WB. **(J-L)** Density calculations for NOX4, PRDX1 and TRX2. Data were expressed as mean ± standard deviation (x ± SD) (n=6). P values were calculated using One-Way ANOVA followed by Fisher’s LSD test. vs control group, *p < 0.05, **p < 0.01; ABX (FD) vs ABX (FD+CHSGP) group, ns*p* > 0.05.

Following antibiotic administration, there was the significant increase in ROS levels (*p* < 0.01) and a decrease in SOD levels (*p* < 0.05) in the gastric tissue mitochondria compared with the control group (**Figures 9A, B**). However, there were no significant differences in ROS and SOD levels between the ABX (FD) and ABX (FD + CHSGP) group (both *p* > 0.05) (**Figures 9A, B**). The co-localization expression intensities of NOX4, PRDX1, and TRX2 were elevated in the ABX, ABX (FD), and ABX (FD + CHSGP) groups compared with the control group (*p* < 0.01 or *p* < 0.05) (**Figures 9C-H**). No significant differences were found between the ABX (FD) and ABX (FD + CHSGP) groups (both *p* > 0.05) (**Figures 9C-H**). Additionally, the ABX, ABX (FD), and ABX (FD + CHSGP) groups showed an increase in NOX4 protein expressions (both *p* < 0.01), a decrease in PRDX1 protein expressions (both *p* < 0.05), and a reduction in TRX2 protein expressions (both *p* < 0.01) compared with the control group (**Figures 9I-L**). However, no significant differences were seen in the protein levels of NOX4, PRDX1, and TRX2 between the ABX (FD) and ABX (FD + CHSGP) groups (both *p* > 0.05) (**Figures 9I-L**). This indicated that the beneficial effect of CHSGP on mitochondrial OS was lost after GM depletion. Consequently, the GM contributed to the antioxidant effects of CHSGP on the mitochondria.

#### 3.7.2 Mitochondrial OS changes in FD rats after the FMT treatment

**Figure 10:**
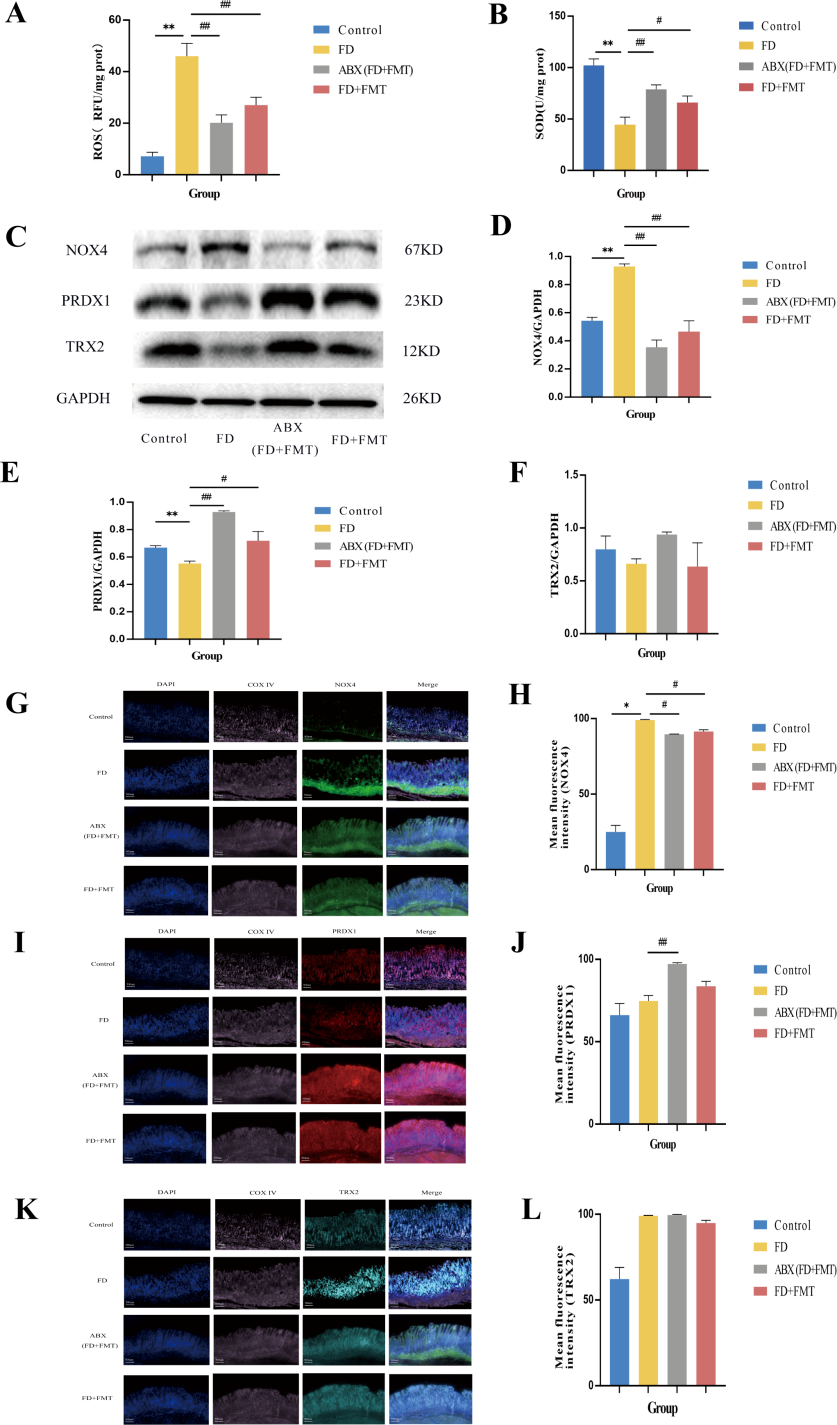
Changes of the mitochondrial OS in FD rats after FMT. **(A)** ROS content analyzed by chemiluminescence. **(B)** SOD content analyzed by colourimetric assay. **(C)** The protein expression levels of NOX4, PRDX1 and TRX2 were analyzed by WB. **(D-F)** Density calculations for NOX4, PRDX1 and TRX2. **(G)** Staining of gastric tissue with the mitochondrial marker COX IV (pink) and the OS marker NOX4 (green). **(H)** Mean fluorescence intensity of NOX4. **(I)** Staining of gastric tissue with the mitochondrial marker COX IV (pink) and the OS marker PRDX1 (red). **(J)** Mean fluorescence intensity of PRDX1. **(K)** Staining of gastric tissue with the mitochondrial marker COX IV (pink) and the OS marker TRX2 (turquoise). **(L)** Mean fluorescence intensity of TRX2. Data were expressed as mean ± standard deviation (x ± SD) (n=6). P values were calculated using One-Way ANOVA followed by Fisher’s LSD test. vs control group, *p < 0.05, **p < 0.01; vs FD group, ^#^ *p* < 0.05, ^##^ *p* < 0.01.

Rats, regardless of GM depletion, received fecal solutions from CHSGP-treated FD rats. Following the FMT treatment in experiment III, there was a significant reduction in mitochondrial ROS levels (*p* < 0.01)and an increase in SOD levels (*p* < 0.05) compared to the FD group (**Figures 10A, B**).Additionally, the NOX4 protein content was notably reduced in the ABX (FD+FMT) and FD + FMT groups (both *p* < 0.01). In comparison, the PRDX1 proteins exhibited markedly elevated (*p* < 0.01 and *p* < 0.05 respectively) (**Figures 10C-E**), while the TRX2 proteins displayed no significant differences between the groups (both *p* > 0.05) compared with the FD group (**Figure 10F**). The ABX (FD + FMT) group showed the stronger antioxidant effect, characterized by reduced NOX4 expression and elevated PRDX1 expression, compared to the FD + FMT group (**Figures 10C-E**). Moreover, there was the reduced co-localization of NOX4 and Cytochrome oxidase IV (COX IV) (*p* < 0.05) and the increased co-localization of PRDX1 and COX IV (*p* < 0.01) compared with the FD group (**Figures 10G-J**). However, there were no statistically significant differences in the co-localization expression of TRX2 and Cytochrome oxidase IV (COX IV) between the ABX (FD+FMT) or FD+FMT groups compared to the FD group (both *p* > 0.05) (**Figures 10K-L**). Therefore, the antioxidant effects of CHSGP on the mitochondria depended on the GM.

### 3.8 CHSGP exerted antioxidant effects by regulating the diversity and composition of the GM in FD rats

#### 3.8.1 Diversity and composition changes of the GM in FD rats after ABX treatment

**Figure 11:**
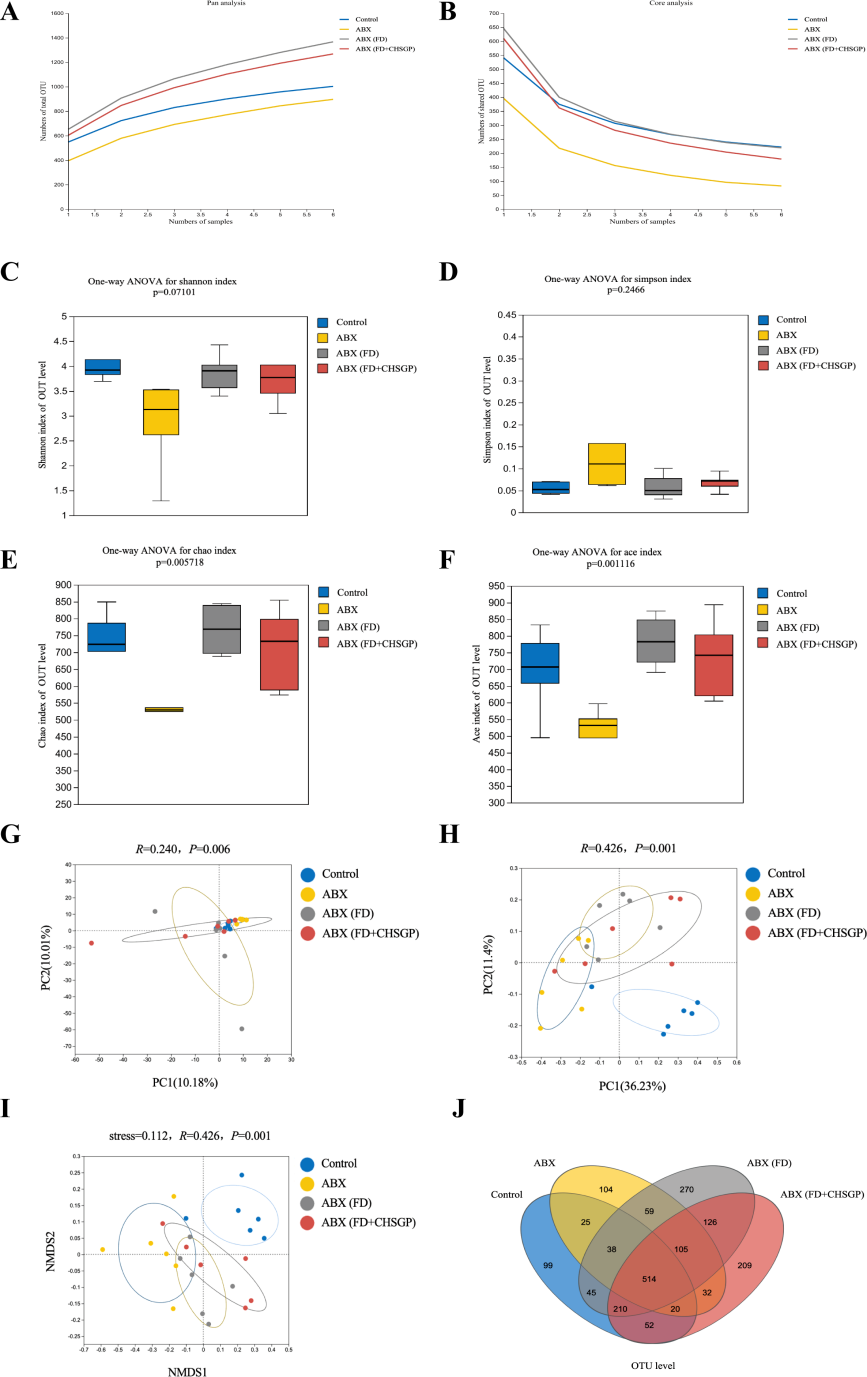
Diversity analysis of the GM of FD rats after ABX. **(A, B)** Pan/Core curve. **(C-F)** Alpha diversity analysis. **(C)** Shannon index analysis. **(D)** Simpson index analysis. **(E)** Chao index analysis. **(F)** Ace index analysis. **(G-I)** Beta diversity analysis. **(G)** PCA analysis. **(H)** PCOA analysis. **(I)** NMDS analysis. **(J)** OTU analysis: venn diagrams. Data were expressed as mean ± standard deviation (x ± SD) (n=6). Differences between samples were compared using the ANOSIM test.

The pan/core curve analysis suggested that the number of sequenced samples adequately met the required criteria (**Figures 11A, B**). The α-diversity analysis showed no statistically significant difference in the Simpson and Shannon indices across groups (both *p* > 0.05) (**Figures 11C, D**). However, the statistically significant difference was observed in the Ace and Chao indices (both *p* < 0.01). This result indicated that ABX could reduce the richness of the microbiota, but ABX (FD) and ABX (FD + CHSGP) could increase the abundance of the microbiota (**Figures 11E, F**). The PCA, PCoA, and NMDS analyses demonstrated significant changes in GM composition in the antibiotic-depleted groups (ABX, ABX (FD), and ABX (FD + CHSGP)) compared to the control group (both *p* < 0.01) (**Figures 11G-I**). The comparative analysis indicated no significant differences in the overall microbiota structure among the ABX, ABX (FD), and ABX (FD + CHSGP) groups (*p* > 0.05) (**Figures 11G-I**). These results indicated that CHSGP administration after the antibiotic treatment did not affect the overall composition of the GM in FD rats. The Venn diagram showed that all four groups collectively shared 514 OTUs, while the control group had 1003 OTUs. In addition, the ABX group contained 838 OTUs, the ABX (FD) group comprised 1369, and the ABX (FD + CHSGP) group included 1270. (**Figure 11J**). The comparison with the control group revealed a drop in microbial OTUs in the ABX group, suggesting the effective depletion of the GM in rats due to ABX treatment.

**Figure 12:**
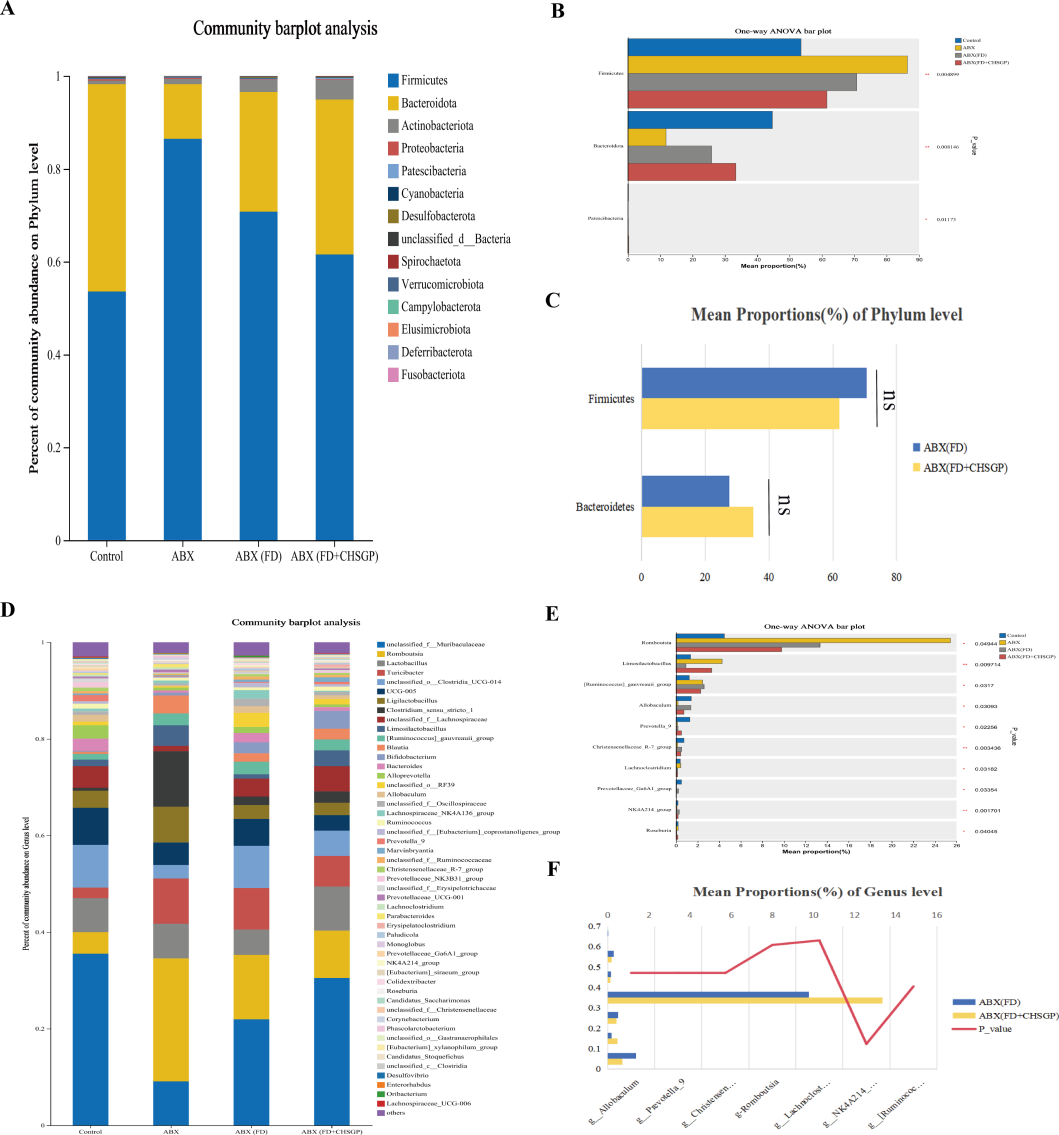
Species composition analysis of the GM of FD rats after ABX. **(A)** Columnar stack diagram of species composition at the phylum level between control, ABX, ABX (FD) and ABX (FD + CHSGP) groups. **(B)** Differential strains at the phylum level between control, ABX, ABX (FD) and ABX (FD + CHSGP) groups. **(C)** Differential strains at the phylum level between ABX (FD) and ABX (FD + CHSGP) groups. **(D)** Columnar stack diagram of species composition at the genus level between control, ABX, ABX (FD) and ABX (FD + CHSGP) groups. **(E)** Differential strains at the genus level between control, ABX, ABX (FD) and ABX (FD + CHSGP) groups. **(F)** Differential strains at the genus level between ABX (FD) and ABX (FD + CHSGP). Data were expressed as mean ± standard deviation (x ± SD) (n=6). P values were calculated using one-way ANOVA to compare multiple groups, and Wilcoxon rank-sum test to compare two groups. *p < 0.05, **p < 0.01, ns, *p* > 0.05.

Ten principal bacterial phyla were identified within the groupings, with Firmicutes and Bacteroidetes being the most dominant, constituting almost 90% of the overall bacterial population (**Figure 12A**). In comparison to the control group, the richness of Bacteroidetes declined (*p* < 0.01), while the richness of Firmicutes increased (*p* < 0.01) in the ABX, ABX (FD), and ABX (FD + CHSGP) groups (**Figure 12B**). However, the above strains exhibited no statistically significant difference between the ABX (FD) and ABX (FD + CHSGP) groups (*p* > 0.05) (**Figure 12C**). These results indicated that ABX could disrupt the GM structure at the phylum level. Meanwhile, CHSGP administration did not affect the GM of FD rats. This indicated that after GM consumption, the regulatory effect of CHSGP on microbial communities at the phylum level was not significant.

Twelve predominant bacterial genera were identified in the groups that accounted for approximately 80% of the overall bacterial population. These included *unclassified_f_Lachnospiraceae*, *norank_f_Muribaculaceae*, *Romboutsia*, *Turicibacter*, *Ligilactobacillus*, *Bifidobacterium*, *Lactobacillus*, and *Alistipes* (**Figure 12D**). Compared with the control group, the abundances of *Allobaculum* (*p* < 0.05), *Prevotella_9* (*p* < 0.05), the *Christensenellaceae_R-7_group* (*p* < 0.01), *Lachnoclostridium* (*p* < 0.05), and the *NK4A214_group* (*p* < 0.01) decreased, while the abundances of *Romboutsia* (*p* < 0.05) and the *[Ruminococcus]_gauvreauii_group* increased (*p* < 0.05) in the ABX, ABX (FD), and ABX (FD + CHSGP) groups (**Figure 12E**). In addition, the abundances of *non-Muribaculaceae*, *Alistipes*, *Romboutsia*, *Turicibacter*, *Bifidobacterium*, *Lactobacillus*, and *Ligilactobacillus* were not statistically significant between the ABX (FD) and ABX (FD + CHSGP) groups (both *p* > 0.05) (**Figure 12F**). These findings showed that ABX could disturb the structure of the GM at the genus level. Accordingly, CHSGP administration did not affect the GM of FD rats. This indicated that CHSGP could lose its ability to regulate the microbiota at the genus level following GM depletion.

#### 3.8.2 Diversity and composition changes of the GM in FD rats after the FMT treatment

**Figure 13:**
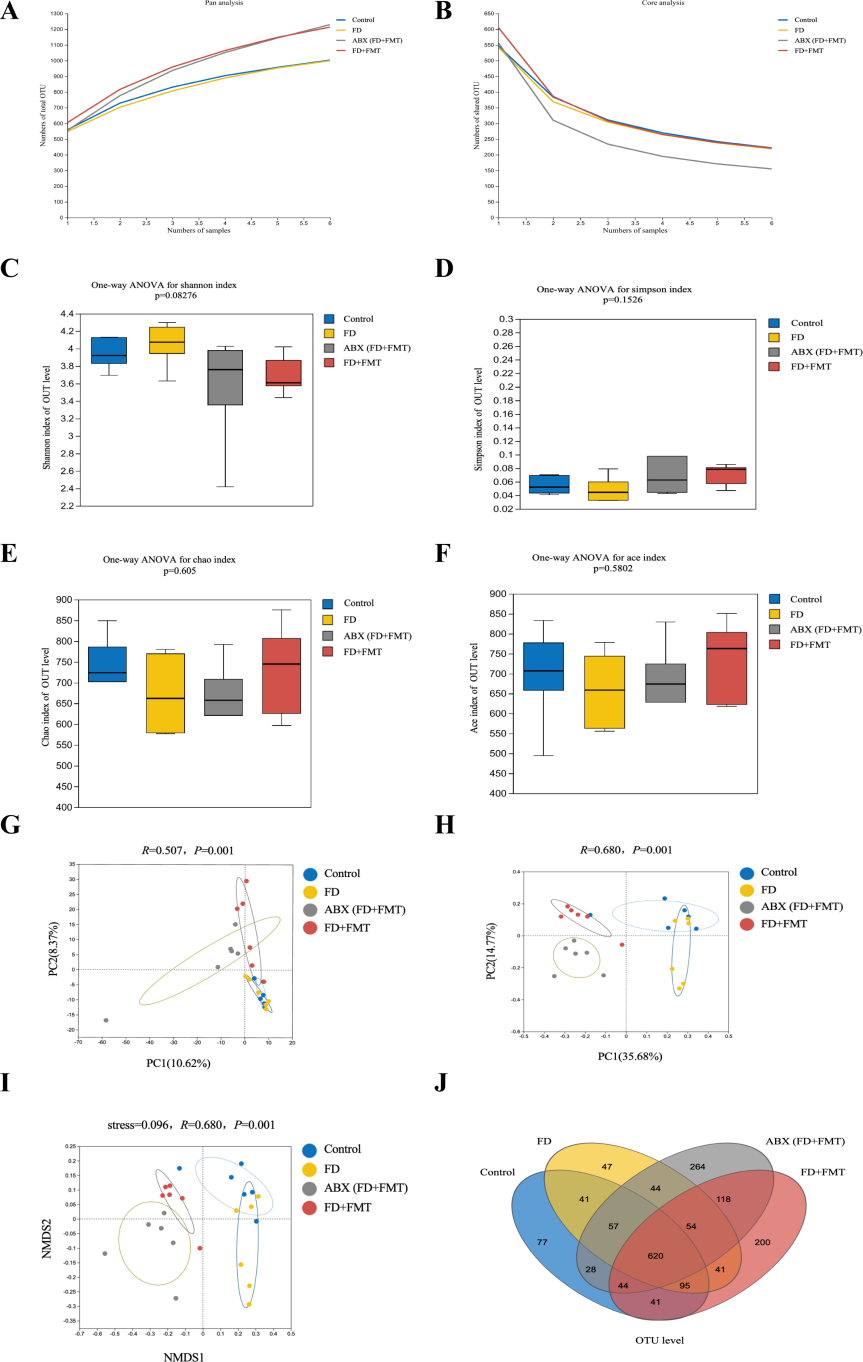
Diversity analysis of the GM of FD rats after FMT. **(A, B)** Pan/Core curve. **(C-F)** Alpha diversity analysis. **(C)** Shannon index analysis. **(D)** Simpson index analysis. **(E)** Chao index analysis. **(F)** Ace index analysis. **(G-I)** Beta diversity analysis. **(G)** PCA analysis. **(H)** PCOA analysis. **(I)** NMDS analysis. **(J)** OTU analysis: venn diagrams. Data were expressed as mean ± standard deviation (x ± SD) (n=6). Differences between samples were compared using the ANOSIM test.

Rats, regardless of GM depletion status, received fecal solutions from CHSGP-treated FD rats. The pan/core curve showed that the sequencing sample number adequately met the established criteria (**Figures 13A, B**). No statistical differences were observed in the Shannon, Simpson, Chao, and Ace indices across the groups in the α-diversity analysis (both *p* > 0.05) (**Figures 13C-F**). This result indicated that the four groups had similar richness and diversity at the α-diversity level. The PCA, PCOA, and NMDS analyses indicated that the GM composition had changed among the control group, FD group, ABX (FD + FMT) group, and FD + FMT group (both *p* < 0.01) (**Figures 13G-I**). The ABX (FD + FMT) and FD + FMT groups had distinct alterations in the GM compared with the FD group. The Venn diagram showed the intersection of the 620 OTUs in all of the groups. The control group had 1003 OTUs, the FD group had 999, the ABX (FD + FMT) group had 1229, and the FD + FMT group had 1213 (**Figure 13J**). This result indicated that the FMT intervention could alter the microbial diversity of rats with FD.

**Figure 14:**
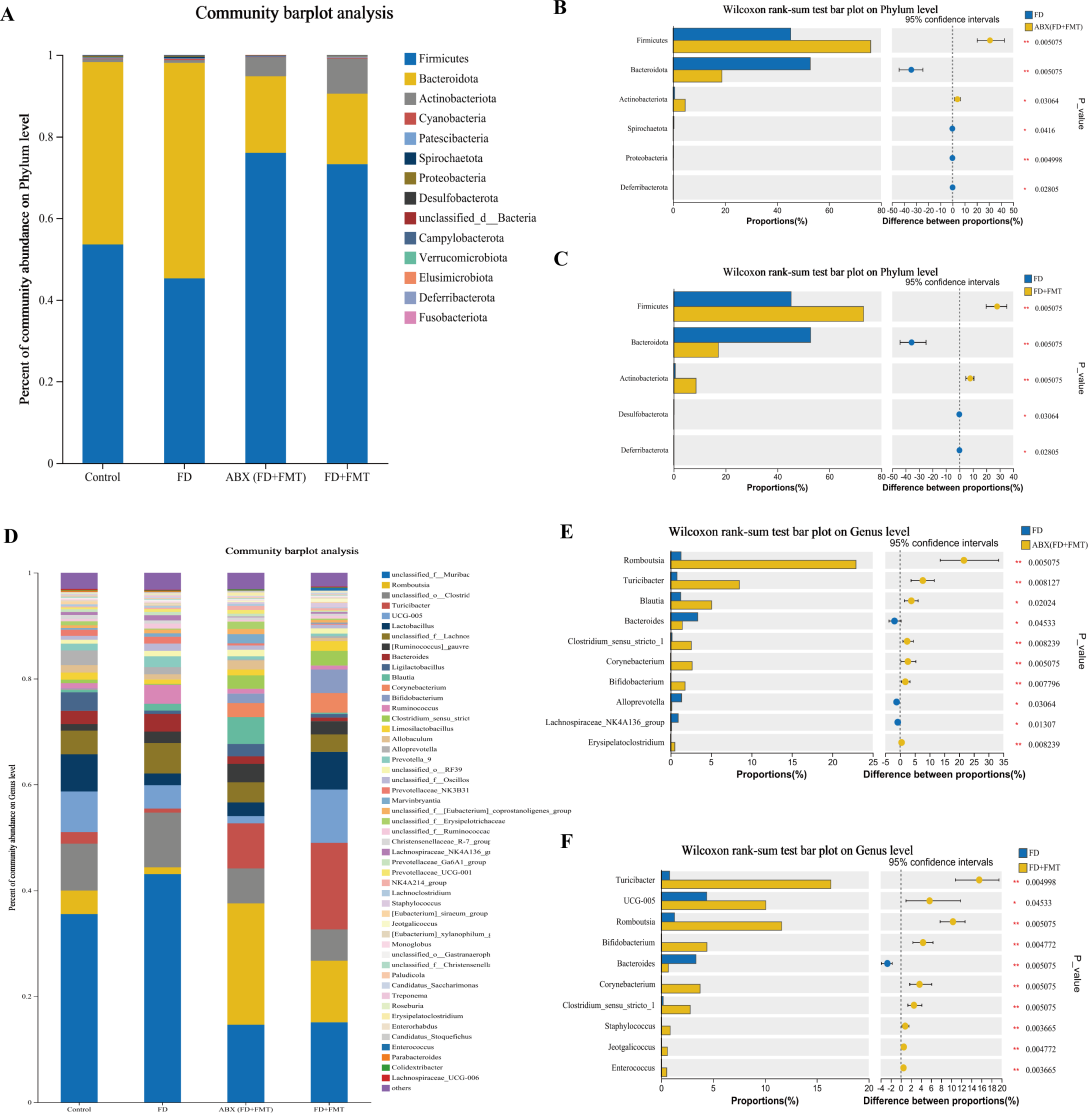
Species composition analysis of the GM of FD rats after FMT. **(A)** Columnar stack diagram of species composition at the phylum level between control, FD, ABX (FD + FMT) and FD + FMT groups. **(B)** Differential strains at the phylum level between FD and ABX (FD + FMT) groups. **(C)** Differential strains at the phylum level between FD and FD + FMT groups. **(D)** Columnar stack diagram of species composition at the genus level between control, FD, ABX (FD + FMT) and FD + FMT groups. **(E)** Differential strains at the genus level between FD and ABX (FD + FMT) groups. **(F)** Differential strains at the genus level between FD and FD + FMT groups. Data were expressed as mean ± standard deviation (x ± SD) (n=6). P values were calculated using Wilcoxon rank-sum test. *p < 0.05, **p < 0.01.

Ten bacterial groups were identified at the phylum level, with Firmicutes, Bacteroidetes, and Actinobacteriota being the most predominant in each group (**Figure 14A**). The GM structures were regulated after the FMT. A decrease in Bacteroidetes (both *p* < 0.01) and an increase in Firmicutes (both *p* < 0.01) and Actinobacteriota (*p* < 0.05 and *p* < 0.01, respectively) were observed in comparison to the FD group. These results indicated that the FMT might influence the composition of GMat the phylum level in FD rats.

Eleven predominant bacterial genera were identified at the genus level, accounting for nearly 80% of the total bacterial population. These genera included *unclassified_f_Lachnospiraceae*, *norank_f_Muribaculaceae*, *Romboutsia*, *Turicibacter*, *Ligilactobacillus*, *Bifidobacterium*, *Lactobacillus*, and *Alistipes*. (**Figure 14D**). The FMT administration promoted changes in the GM at the genus level in FD rats. Compared to the FD group, the ABX (FD + FMT) group showed a notable reduction in the levels of *Bacteroides* (*p* < 0.05), *Alloprevotella* (*p* < 0.05), and the *Lachnospiraceae_NK4A136_group* (*p* < 0.05), whereas *Romboutsia* (*p* < 0.01), *Bifidobacterium* (*p* < 0.01), *Turicibacter* (*p* < 0.01), *Corynebacterium* (*p* < 0.01), *Clostridium_sensu_stricto_1* (*p* < 0.01), and *Blautia* rose (*p* < 0.05) (**Figure 14E**). The FD + FMT group had a decrease in *Bacteroides* (*p* < 0.01), while *Romboutsia* (*p* < 0.01), *Bifidobacterium* (*p* < 0.01), *Turicibacter* (*p* < 0.01), *UCG-005* (*p* < 0.05), *Corynebacterium* (*p* < 0.01), and *Clostridium_sensu_stricto_1* increased (*p* < 0.01) (**Figure 14F**). These results indicated that the FMT might influence the compositions of GM at the genus level in FD rats.

### 3.9 FMT regulated the GM to alleviate OS

**Figure 15:**
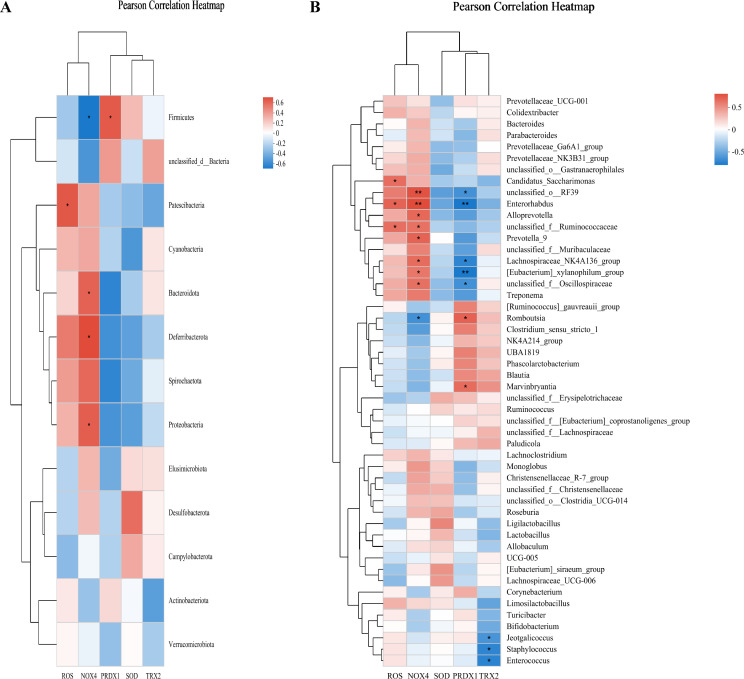
FMT regulated the GM to alleviate OS. **(A)** Heatmap of the correlation between the differential microbiota at the phylum level among the control, FD, and FD + FMT groups and the indicators of OS. **(B)** Heatmap of the correlation between the differential microbiota at the genus level among the control, FD, and FD + FMT groups and the indicators of OS. Average was used for both clinical factors and species hierarchical clustering methods. We calculated the correlation between variables using the pearson correlation coefficient. *p < 0.05, **p < 0.01.

Pearson’s correlation analysis was employed to evaluate the association across the GM and OS indicators (ROS, SOD, NOX4, TRX2, and PRDX1) in the control, FD, and FD + FMT groups. At the phylum level, Firmicutes was positively correlated with PRDX1 (*p* < 0.05) and negatively correlated with NOX4 (*p* < 0.05). Patescibacteria was positively correlated with ROS (*p* < 0.05). Bacteroidota, Deferribacterota, and Proteobacteria were positively correlated with NOX4 (both *p* < 0.05) (**Figure 15A**). At the genus level, *Enterorhabdus* was positively correlated with ROS (*p* < 0.05) and NOX4 (*p* < 0.01), while it was negatively correlated with PRDX1 (*p* < 0.01). *Romboutsia* was positively correlated with PRDX1 (*p* < 0.05), while it was negatively correlated with NOX4 (*p* < 0.05). *Unclassified-o-RF39*, the *Lachnospiraceae-NK4A136-group*, the *[Eubacterium]-xylanophilum-group*, and *Unclassified-f-oscillospiraceae* exhibited positive correlations withNOX4 (*p* < 0.01, *p* < 0.05, *p* < 0.05, and *p* < 0.05, respectively), demonstrating negative correlations with PRDX1 (*p* < 0.05, *p* < 0.05, *p* < 0.01, and *p* < 0.05, respectively). *Alloprevotella* and *Prevotella-9* showed positive correlations with NOX4 (both *p* < 0.05) (**Figure 15B**).

## 4 Discussion

Previous studies emphasized that dyskinesia of the GI tract and disorders of GM were key pathological mechanisms of FD (4). TCM significantly enhanced mitochondrial function, GI motility improvements, and GM regulation in FD (5, 10, 11). We found that CHSGP relied on the GM to relieve mitochondrial OS damage in gastric tissue, hence enhancing gastrointestinal motility. This investigation was divided into three portions. We first explored the impact of CHSGP on OS-related markers (ROS, SOD, NOX4, PRDX1, and TRX2) and GM in FD rats. We then implemented GM depletion using a four-antibiotic cocktail to evaluate whether the effect of CHSGP on OS was GM-dependent in FD rats. Finally, we examined the effect of the fecal microbiota from rats treated with FD + CHSGP on the OS and GM under different GM conditions. The schematic representation of the findings and mechanisms is illustrated in **Supplementary Figure S3** (By Figdraw).

The emergence of numerous GI diseases is associated with OS. These diseases include neurogastrointestinal disorders, inflammatory bowel disease, peptic ulcers, and GI malignancies (32). OS arises when the generation of ROS exceeds the capacity of cells to eliminate or neutralize them via antioxidants (32). The NOX family, known as NOXs, consists of transmembrane proteins that facilitate electron transfer across biological membranes to catalyze the reduction of oxygen to superoxide (O_2−_) (33). Pathological stimuli such as inflammation, hypoxia, and ischemia can induce the expression of NOX4, subsequently promoting the production of ROS (33). Antioxidant mechanisms involve enzymes that produce reduced forms of antioxidants and ROS-interacting enzymes, including peroxidases, SOD, and oxidoreductases. These mechanisms are crucial for the regulation of ROS levels *in vivo*. SOD serves as the principal defensive mechanism against diseases or damage caused by ROS. SOD comprises a class of metalloenzymes that uses metal ions in their active sites to catalyze the conversion of ROS into molecular oxygen and H_2_O_2_ for further use in different biological processes. The PRDX oxidoreductase contains an ionized thiol that converts ONOO− and other peroxides to H_2_O_2_ by oxidizing the conserved thiolate to -SOH intermediates. These can interact with resolving cysteine thiols (-SH). The Trx system comprises Trx and its associated thioredoxin reductase (TR). TR uses NADPH as an electron donor to facilitate the reduction and reconstruction of the dithiol active site in oxidized Trx. Mishina (34) stated that the Trx system was crucial for establishing an intracellular H_2_O_2_ gradient creation and for inhibiting the diffusion of H_2_O_2_ throughout the cytoplasm. Experiment I showed that the mitochondrial structure of the ICC is compromised, along with alterations in OS indicators in FD group. These changes suggested that dysregulation of gastric motility was associated with OS damage to gastric tissue mitochondria in FD. In addition, research has linked OS development to peptic ulcer disease and non-ulcer dyspepsia (35). After CHSGP treatment, all of the mentioned pathological changes were reversed. This suggested that CHSGP alleviated mitochondrial OS in the stomach tissues of FD rats. Wei Kangning also treated indigestion using antioxidant methods (5). Except for FD, the common functional gastrointestinal disorders (FGIDs) included functional constipation and irritable bowel syndrome (IBS). Some studies have shown that TCM can treat these conditions by alleviating OS. For slow-transmitting constipation, the Zhizhu decoction can alleviate intestinal inflammation and OS by activating the SIRT1/FoxO1 antioxidant signaling pathway in the colon (36). STW5 reduced the 5-hydroxytryptamine (5-HT) signaling pathway in the brain-gut axis and combated stress-induced OS to facilitate the treatment of IBS (37).

Microbiota dysbiosis refers to a disruption in the microbiota structure and function, affected by environmental and host-related factors (38). This disruption surpasses the resilience and recovery capacity of microbial ecosystems, leading to GI disorders (38). Experiment I revealed that the FD group exhibited microbial dysbiosis. The literatures also confirmed that the microbial disorders that were investigated in this study contributed to FD development. For example, 16S rRNA miseq sequencing showed a link between dyspepsia symptoms and the Firmicutes and Bacteroides levels (7). The *Ligilactobacillus* and *Bifidobacterium* genera are recognized as beneficial bacteria that can stimulate the protective response of the host immune system (39, 40). Studies have shown the connection between the development of functional GI disorders and the absence of these bacteria (41, 42). This study revealed that FD might result in a reduction in Firmicutes, *Ligilactobacillus*, and *Bifidobacterium* while simultaneously elevating levels of Bacteroidetes, *Erysipelotrichaceae_UCG-003*, and *Collinsella*. The CHSGP decoction modulated the GM of FD by decreasing the levels of Bacteroidetes, *Prevotellaceae_UCG-001*, *Colidextribacter*, *Alistipes*, and *Akkermansia*, while increasing the levels of Firmicutes, *UCG-005*, *Oribacterium*, *Negativibacillus*, and *Bifidobacterium*. Moreover, the literature review suggested that the child compound endothelium corneum enhanced Firmicutes and diminished Bacteroidetes in rats, playing a therapeutic role in FD (7). Chinese medicine can treat other FGIDs by affecting the GM. The XiaoChengQi decoction alleviated slow-transmitting constipation by increasing the relative abundances of *Lactobacillus*, *Muribaculaceae*, the *Prevotellaceae_NK3B31_group*, and *Roseburia* in feces and decreasing the relative abundances of the *Lachnospiraceae_ NK4A136_group* and *Desulfovibrio* (43). Tong-Xie-Yao-Fang has demonstrated efficacy in alleviating symptoms of irritable bowel syndrome with diarrhea (IBS-D) by enhancing the GM diversity and modifying the relative abundance of *Akkermansia* and *Clostridium sensu stricto 1* within gut microbial communities (44).

Microbial changes can affect the OS. Inflammation generated by *Helicobacter pylori* alters the thiol-disulfide equilibrium in the cellular redox system, a pivotal process in the pathogenesis of non-ulcer dyspepsia (45). Several studies have indicated that the GM could induce OS in various clinical contexts, including neurodegenerative diseases (46), atherosclerosis (47), and type 2 diabetic (48). Therefore, we can use drugs and probiotics to regulate the GM to alleviate OS (15). In Experiment I, we performed the correlation analysis of the differing microbial communities and OS indicators among the control, FD, and FD + CHSGP groups to investigate whether the CHSGP decoction could alleviate OS by modulating the GM. The results revealed some specific microbial species linked to OS at the phylum and genus levels. To evaluate the influence of GM depletion on the antioxidant properties of the CHSGP decoction, the rats were treated with four antibiotics to deplete their GM and observe changes in the OS-related indices (Experiment II). There were significant differences in the GM compositions between the control and ABX groups, indicating the effectiveness of antibiotics in depleting the microbiota. We compared OS-related indicators in the ABX (FD) and ABX (FD + CHSGP) groups in Experiment II. The results indicated that the CHSGP decoction failed to adequately modulate OS after the microbiota depletion. To verify that CHSGP relied on the GM to alleviate OS, we administered a fecal microbiota solution from the FD + CHSGP group to monitor changes in the OS indicators. The findings showed that the FMT treatment alleviated OS and repaired the mitochondrial structure damage. This result confirmed that CHSGP relied on the GM to reduce OS in FD rats. Interestingly, the ABX (FD + FMT) group showed a more significant protective effect against mitochondrial OS than the FD + FMT group. This result was likely attributable to the interference from the natural microbiota in rats. Microbiota sequencing analysis showed changes in the microbiota composition in the ABX (FD + FMT) and FD + FMT groups compared with the FD group. We conducted the correlation analysis between microbial species differences across various groups and OS markers. The results indicated that the FMT increased the abundance of microbiota that inhibited OS. These bacteria included Firmicutes and *Romboutsia*. The FMT also suppressed microbiota that promoted OS, including Bacteroidota, Deferribacterota, *Enterorhabdus*, *Alloprevotella*, and *Prevotella-9*. Studies have proven that the above bacterial changes affect the OS process. For instance, researchers have demonstrated that Firmicutes affects glutathione production by modulating the activity of the essential enzyme glutamate-cysteine ligase catalytic subunit (Gclc) (49). Additionally, these bacteria suppress ROS accumulation by activating the cAMP response element-binding pathway (49). *Group A Streptococcus* belongs to Firmicutes, and its metabolic byproducts, including SOD, NADH oxidase, TRX, nicotinamide adenine dinucleotide oxidase, alkyl hydroperoxide reductase, and glutathione reductase, exhibit antioxidant properties (50). Furthermore, *Group A Streptococcus* possesses proteins that facilitate DNA repair and the restoration of proteins damaged by ROS. It also contains metal ion transporters that indirectly control metal homeostasis to fight OS (51). *Bacteroides fragilis* is an anaerobic bacterium that is part of the Bacteroides genus found in the human intestine. Research has indicated that purified *Bacterotoxin fragile* triggers a rise in spermine oxidase (SMO) levels in HT29/c1 and T84 colonic epithelial cells. This causes ROS production in an SMO-dependent manner (52). Although there are no particular investigations on the impact of *Romboutsia* on OS, inflammation in the pig colon has demonstrated a reduction in both *Romboutsia* levels and total antioxidant capacity activity (53). This suggested a potential correlation between the two. Deferribacterota are anaerobic bacteria capable of oxidizing many complex organic compounds and organic acids. They are classified as sulfate-reducing bacteria, and researchers have confirmed their phylogenetic position as an incomplete oxidizer based on the dissimilatory sulfate reductase phylogeny (54). An increase in *Enterorhabdus* is associated with OS-related diseases such as colitis (55) and diabetes (48), and thus, this genus is often considered an OS-promoting strain. *Alloprevotella* and *Prevotella-9* are Gram-negative anaerobic bacteria within the Prevotella genus, characterized by low tissue redox potentials (35). Santos (56) used matrix-assisted laser desorption/ionization time-of-flight (MALDI-TOF/TOF) tandem mass spectrometry to reveal that the differentially regulated protein sequences in *Prevotella* intermedia are associated with antioxidant and redox regulatory functions. Our findings demonstrated that CHSGP might alleviate mitochondrial OS in the gastric tissue of FD rats by decreasing the abundances of OS-promoting strains (Bacteroidota, Deferribacterota, *Enterorhabdus*, *Alloprevotella*, and *Prevotella-9*) and increasing the abundances of OS-inhibiting strains (Firmicutes and *Romboutsia*).

Consistent with the findings of the current investigation, researchers have found that the Shen-Ling-Bai-Zhu-San therapy modulates microbial structure, leading to the enhancement of energy metabolic pathways and the reduction in OSto treat FD (57). Furthermore, the literature has proven that herbal medicines can treat other FGIDs by modulating the relationship between OS and the GM. Network pharmacology and molecular docking analyses have revealed that the rhubarb peony decoction may provide a comprehensive therapeutic effect on the overlapping syndromes of ulcerative colitis and IBS through a multi-component, multi-target, multi-pathway biological mechanism addressing OS, immune dysfunction, and gut microbial dysbiosis (58). The Simo decoction can increase the abundance of beneficial GM to relieve OS and treat constipation (59).

The mechanism by which the CHSGP decoction restored GI movement may be associated with the alleviation of OS due to GM regulation. Specifically, the CHSGP decoction enhanced the abundances of OS-inhibiting strains (Firmicutes and *Romboutsia*) while decreasing the abundances of OS-promoting strains (Bacteroidota, Deferribacterota, *Enterorhabdus*, *Alloprevotella*, and *Prevotella-9*). Future research can be pursued from two perspectives. Firstly, we will extract and purify the chosen core microbial communities, such as the OS-inhibitory strains Firmicutes and *Romboutsia*, to assess their efficacy and safety in treating FD rats. The data gained will be implemented in the clinical treatment of patients with FD to assess their therapeutic benefits. Secondly, in terms of fundamental research, the GM exerts its effects through metabolic products; therefore, we will subsequently investigate the specific mechanisms by which CHSGP counteracts mitochondrial OS in ICC from the metabolomics perspective. On the other hand, microbial dysbiosis underlies the symptoms associated with FGIDs (60). This study also inspires us to explore the role of the microbiota in the pathogenesis and treatment of FGIDs, based on the symbiotic relationship between the microbiota and the host.

However, this study has some limitations. First, the model reproduced the diminished gastrointestinal motility characteristic with FD. However, the pathological mechanisms of FD in humans encompass not only delayed gastric emptying but also impaired gastric accommodation, hypersensitivity to gastric distension, and abnormalities in the gut-brain axis (25). Therefore, this study does not fully represent human FD. Second, differences in genetic background can affect the therapeutic applicability of findings derived from animal studies. Third, microbiota dysbiosis is a central mechanism underlying the occurrence of FD. Broad-spectrum antibiotics can reduce the GM, which not only affects the OS of ICC but may also influence GI motility through other mechanisms. For instance, the administration of ampicillin in mice resulted in the decrease in the number of enteric neurons within the colonic muscle tissue, thereby impairing GI motility (61). This study specifically evaluated OS, and we cannot rule out the possibility that the GM may contribute to the development of FD through other pathways.

## Data Availability

The data presented in the study are deposited in the Figshare repository, accession number 10.6084/m9.figshare.28389212.

## Supplementary material

Supplementary Figure 1 Experimental operation diagram.

Supplementary Figure 2 Experimental procedure timeline diagram.

Supplementary Figure 3 Mechanistic diagram.
