# Supplementary figures and images for: Chaihushugan powder regulates the gut microbiota to alleviate mitochondrial oxidative stress in the gastric tissues of rats with functional dyspepsia
Source: Front Immunol. 2025 Feb 18;16:1549554. doi: 10.3389/fimmu.2025.1549554 (PMC11876139; doi:10.3389/fimmu.2025.1549554)

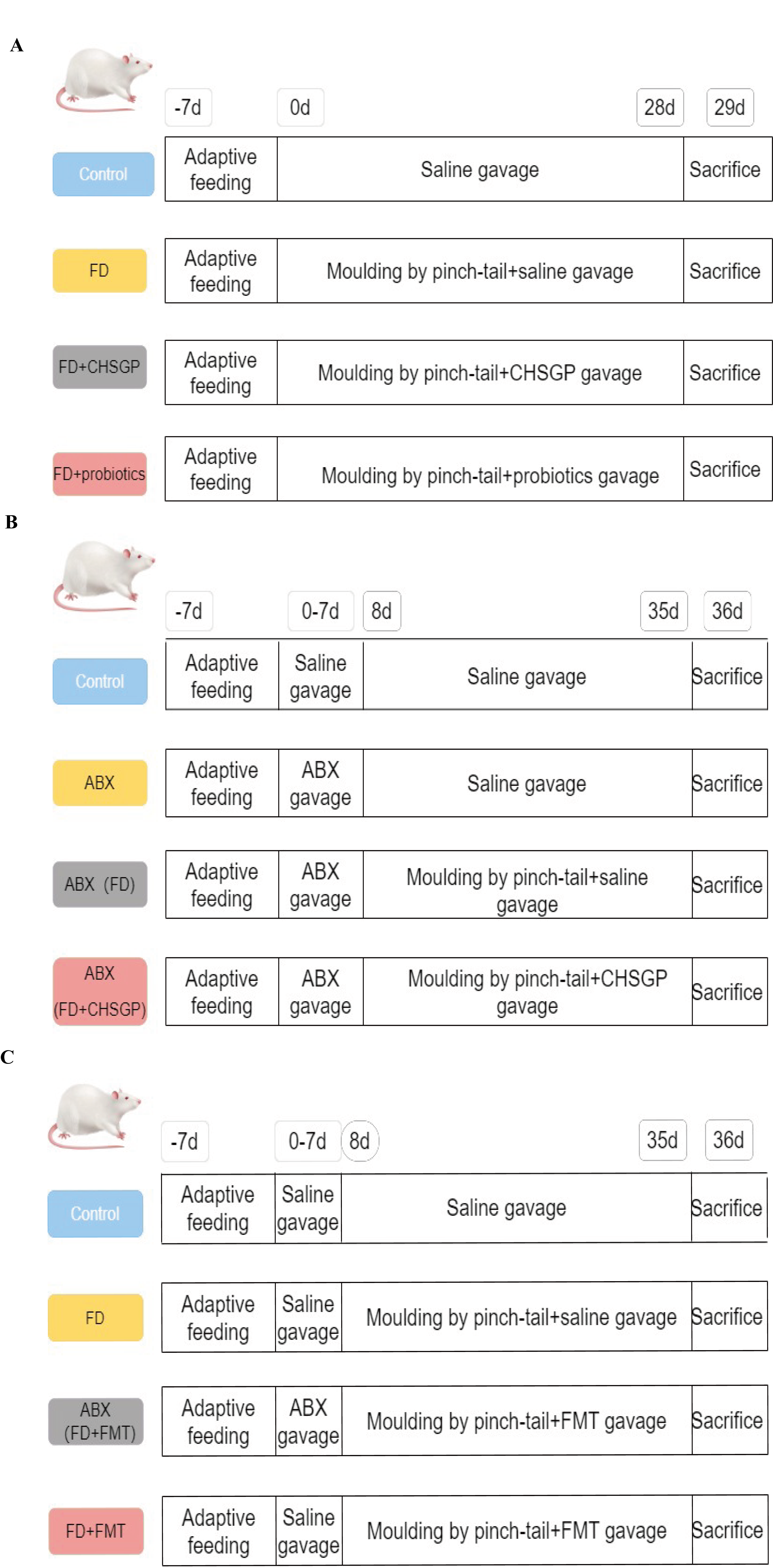

Supplement: Supplementary Figure 1 — Experimental operation diagram. [file Image1.tif]

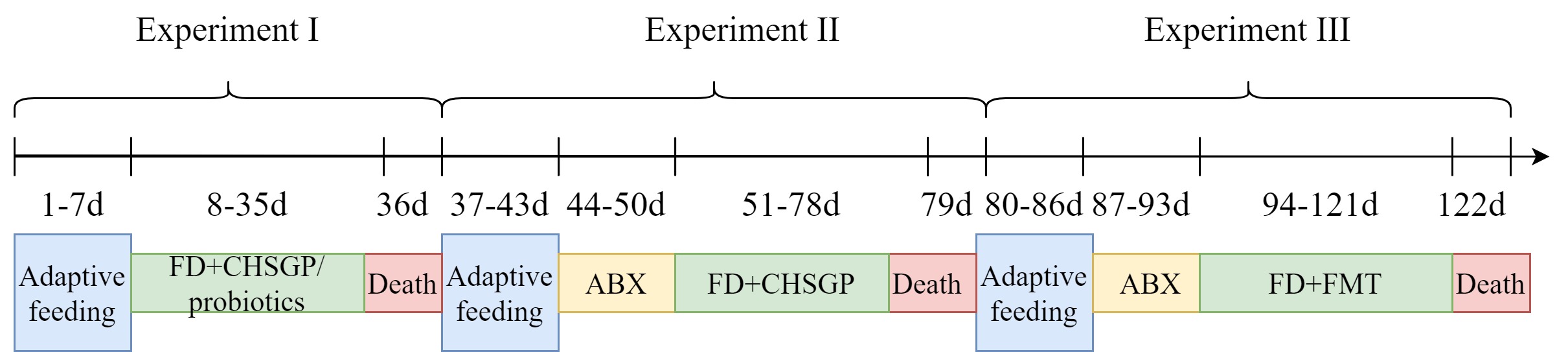

Supplement: Supplementary Figure 2 — Experimental procedure timeline diagram. [file Image2.jpeg]

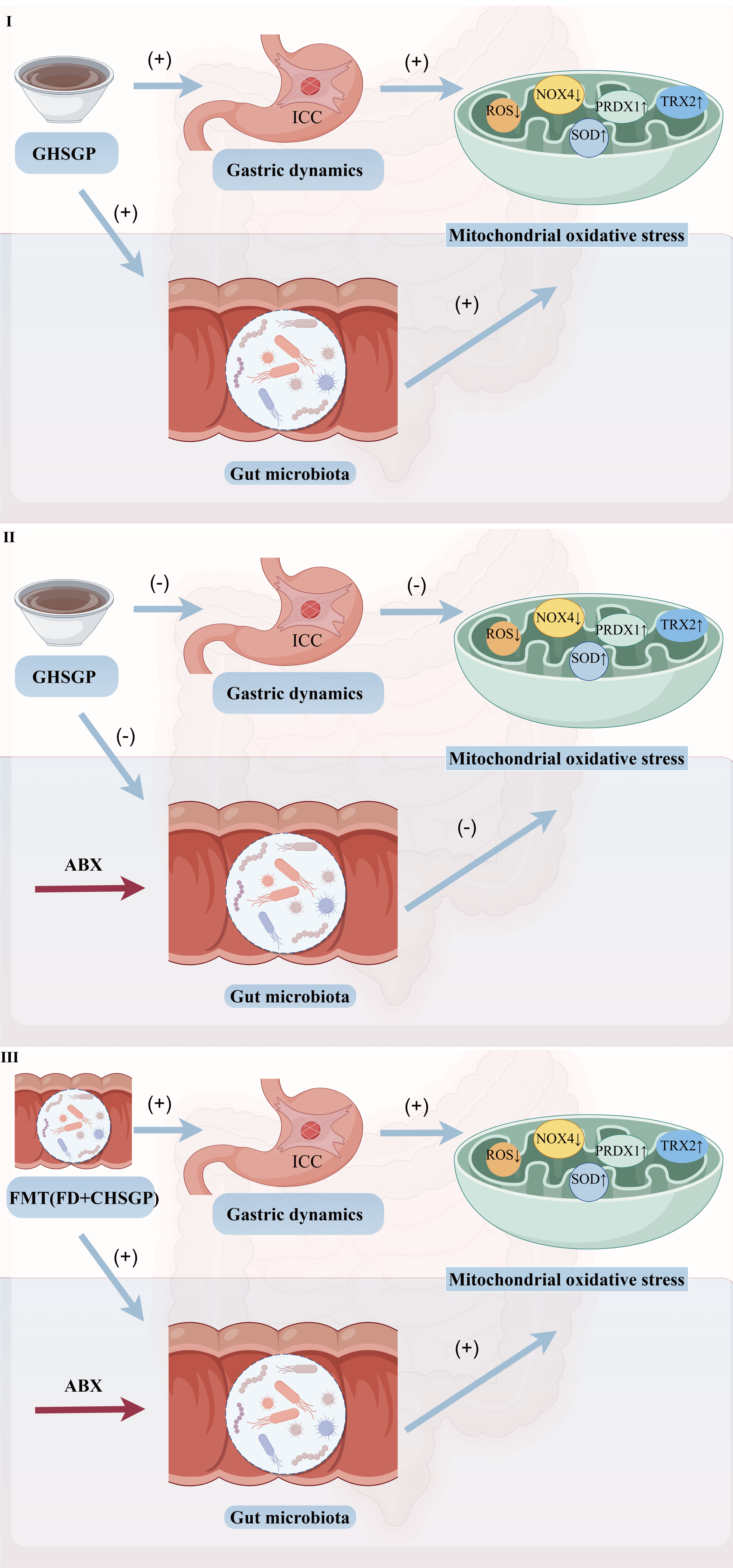

Supplement: Supplementary Figure 3 — Mechanistic diagram. [file Image3.tif]
